# The Effect of Increased Plant Protein Intake on the Lipid Profile of Chronic Kidney Disease Patients: A Meta-Analysis of Controlled Clinical Trials

**DOI:** 10.3390/nu17091408

**Published:** 2025-04-23

**Authors:** Ioanna Papaodyssea, Areti Lagiou, Ioanna Tzoulaki, Elisavet Valanou, Androniki Naska

**Affiliations:** 1 Department of Hygiene, Epidemiology and Medical Statistics, School of Medicine, National and Kapodistrian University of Athens, 115 27 Athens, Greece; ioannnapap92@gmail.com (I.P.); evalanou@med.uoa.gr (E.V.); 2 Department of Public and Community Health, School of Public Health, University of West Attica, 122 43 Athens, Greece; alagiou@uniwa.gr; 3 Department of Epidemiology and Biostatistics, School of Public Health, Imperial College London, London SW7 2AZ, UK; itzoulaki@bioacademy.gr; 4 Systems Biology, Biomedical Research Institution Academy of Athens, 115 27 Athens, Greece

**Keywords:** chronic kidney disease, CKD, dialysis, plant protein, soy protein, total cholesterol, LDL cholesterol, triglycerides, HDL cholesterol, Apolipoprotein A, ApoA, Apolipoprotein B, ApoB, meta-analysis

## Abstract

**Background/Objectives:** Chronic kidney disease (CKD) is associated with increased mortality, with cardiovascular disease (CVD) being the primary cause of death. Proper lipid regulation may reduce CVD risk and slow CKD progression. While there is evidence that a higher plant protein intake could ameliorate lipid levels in the general population, the effects of this dietary regimen within the CKD population remain uncertain, with studies providing conflicting results. We aim to investigate the impact of increased plant protein intake on the lipid levels of CKD patients. **Methods:** Two electronic databases (PubMed, Scopus) were reviewed for controlled clinical trials assessing the effect of increased plant protein intake versus the usual CKD animal-based diet in CKD patients, published until June 2024. **Results:** Eleven trials, encompassing 248 patients, were included in this meta-analysis. Overall, compared to the usually recommended CKD diet, increased plant protein intake was associated with statistically significant reductions in total cholesterol (−24.51 mg/dL, 95% CI −40.33, −8.69), low-density lipoprotein (LDL) (−21.71 mg/dL, 95% CI −38.32, −5.1), triglycerides (− 21.88 mg/dL, 95% CI −35.34, −8.40), and Apolipoprotein B levels (−11.21 mg/dL, 95% CI −18.18, −4.25). No significant changes were observed in high-density lipoprotein (HDL) (0.09 mg/dL, 95% CI −1.82, 1.99) and Apolipoprotein A levels (0.04 mg/dL, 95% CI −7.14, 7.21). **Conclusions:** Increased plant protein intake, mainly from soy, reduces total cholesterol, LDL, triglycerides, and ApoB in adult CKD patients. Further research is needed to assess these effects in dialysis patients and explore non-soy plant sources.

## 1. Introduction

Chronic kidney disease (CΚD) is a significant contributor to global morbidity and mortality, with the total number of deaths per year ranging from 5 million to 11 million [1]. Deaths due to CKD increased by 50% from 2000 to 2010, and by 2040, it is predicted to be the fifth most common cause of years of life lost (YLL), surpassing other major drivers of early mortality, such as diabetes mellitus [2,3,4]. Cardiovascular disease (CVD) is the leading cause of death among this population, while CKD is increasingly recognized as an independent risk factor for cardiovascular events [5,6,7]. Compared to the general population, patients with CKD stage 3 (estimated glomerular filtration rate 30–59 mL/min/1.73 m^2^) have an approximately two-fold risk of dying from CVD, which becomes three-fold at stage 4 (15–29 mL/min/1.73 m^2^) [8].

Aside from traditional cardiovascular risk factors, which are highly prevalent across all stages of CKD, separate pathways have been identified as possible links between the two entities. The pathological metabolism of calcium and phosphorus, impaired production of Klotho, and increased levels of fibroblast growth factor 23 (FGF 23) contribute to diffuse myocardial fibrosis and diastolic dysfunction [9,10]. Additionally, endothelial dysfunction and plaque formation seem to be aggravated by the highly atherogenic lipidemic profile of CKD patients. This profile is characterized by an increase in triglycerides, very low-density lipoprotein (VLDL), and Lp(a), while high-density lipoprotein (HDL) levels are remarkably low. Low-density lipoprotein (LDL) levels appear normal, but oxidized—thus more atherogenic—levels of LDL are increased [11,12,13,14,15].

Pharmacological or dietary interventions for lipid regulation among CKD patients are thus being extensively researched. The latest nutritional guidelines from the American Heart Association concerning cardiovascular risk reduction in the general population emphasize the importance of protein source, recommending that the majority of protein consumed should be of plant origin [16]. However, the corresponding guidelines for CKD patients do not specify a preferred plant protein source due to insufficient evidence [17].

In a systematic review published in 2014, which included nine randomized controlled trials (RCTs) with 197 participants, the effects of soy protein on kidney function and lipid levels in pre-dialysis CKD patients were investigated [18]. The findings suggested that soy protein had a beneficial effect on serum creatinine, phosphorus, total cholesterol (TC), and triglyceride (TG) levels. However, the reduction in total cholesterol and triglycerides was not statistically significant. To further investigate this relationship, we conducted a meta-analysis on the effect of plant protein on the lipid profile of CKD patients across all stages, taking into account participants’ baseline characteristics and the methodological aspects of the included studies.

## 2. Materials and Methods

This meta-analysis has been registered in PROSPERO (registration number CRD42022331268), and the results are reported in accordance with the Preferred Reporting Items for Systematic Reviews and Meta-Analyses (PRISMA) statement, as well as the standards of the Cochrane Handbook for Systematic Reviews of Interventions [19,20].

### 2.1. Literature Search Strategy 

The PubMed and Scopus databases were searched using appropriate algorithms tailored for each database. The following keywords, along with relevant variations, were used: “Chronic Kidney Disease”, “Glomerular Filtration Rate”, “Proteinuria”, “Renal Replacement Therapy”, “plant protein”, “soy protein”, “vegan protein”, and “animal protein” The detailed search strategy, along with the Boolean operators used, can be found in the Supplementary Materials. Additionally, the bibliographies of included studies were reviewed to identify relevant studies that were not retrieved during the initial search. A thorough search of the gray literature (relevant conference proceedings, clinical trial registries, and Google Scholar) was also conducted using the same keywords. All manuscripts (original studies published in journals or as conference proceedings) published before 22 June 2024 were considered.

### 2.2. Eligibility Criteria and Study Selection

Controlled clinical trials (of either parallel or crossover design) evaluating the impact of increased plant protein intake on the lipid profile of CKD patients were deemed eligible. The specific inclusion criteria are as follows: (1) adult patients diagnosed with CKD at any stage, as defined by KDIGO/KDOQI guidelines [21]; (2) an intervention involving an increase in plant protein intake through dietary modifications or daily supplements, maintained for at least four weeks; (3) a control group following the standard CKD diet, with less than 50% of total protein intake derived from plant sources; and (4) lipid profile indicators (including non-HDL, VLDL, Apolipoprotein A, Apolipoprotein B, and Lp(a)) reported as mean values before and after the intervention. Studies that were not published in English, as well as studies in which participants had indications of acute kidney injury or were renal transplant recipients, were excluded. Screening of the studies identified in the initial search was carried out independently by two authors (IP and AN). Disagreements were resolved via consensus or, if necessary, after discussion with a third author (EV).

### 2.3. Data Extraction and Risk of Bias Assessment

The following data were extracted from each of the eligible studies: name of the first author, year of publication, country of origin, study type, number of participants in each group, age, sex, CKD stage of participants, cause of CKD, percentage or amount of plant protein administered to the intervention arm, type of plant protein, percentage or amount of animal protein administered to the control arm, total daily intake of protein for each group, duration of the intervention, existence of a washout period in crossover trials, baseline lipidemic status, use of lipid-lowering agents and outcome measures (mean ± SD before and after the intervention, as well as the standard deviation of the difference in means). Values expressed as mmol/L were converted to mg/dL (by multiplying either by 38.67 for TC, LDL, and HDL, or by 88.57 for TG). In four studies [22,23,24,25] in which the published data required for the pooled analysis were inadequate, the authors were contacted via email, but no responses were received.

For the calculation of the pooled effect size, the difference in mean values before and after the intervention and their standard deviations were used. If the standard deviations were not provided, the method proposed by Higgins et al. [19], which imputes the missing standard deviation of the difference in means from the mean value of the available standard deviations, was employed. However, standard deviations of the difference in means before and after the intervention were available for less than 50% of the studies; therefore, mean values after the intervention and their standard deviations were used for the calculation of the pooled effect size. Sensitivity analyses were conducted to assess whether the pooled effect sizes were impacted by the imputation of the standard deviations.

To assess the internal validity of the studies, the Cochrane Rob2 tool was used for randomized controlled trials, and the ROBINS-I tool was used for non-randomized controlled interventions [26,27]. Two authors (IP and AN) worked independently, and in cases of disagreement, consensus was reached with the involvement of a third author (EV). The five domains through which bias could be introduced are as follows: (1) bias arising from the randomization process, (2) bias due to deviations from intended interventions, (3) bias due to missing outcome data, (4) bias in the measurement of the outcome, and (5) bias in the selection of the reported result. Each domain was categorized as having a high or low risk of bias, or as raising some concerns. Consequently, based on these categorizations, each study was deemed to have a low risk, high risk, or some concerns regarding the risk of bias [26].

### 2.4. Statistical Analysis

The meta-analysis was conducted using RevMan 5.4 (Review Manager (RevMan) [Computer program]. Version 5.3. Copenhagen: The Nordic Cochrane Centre, The Cochrane Collaboration, 2014). The effect sizes were calculated using the inverse variance method under a random effects model since there was significant heterogeneity between the studies (related to differences in the study population, study design, and intervention). All data were continuous and pooled as weighted mean differences (MD) with 95% confidence intervals (CI). Heterogeneity was also assessed using the *I*^2^ statistic. Subgroup analyses based on CKD stage, study design, percentage of plant protein compared to total protein, risk of bias, and imputation of the standard deviations were carried out. Leave-one-out sensitivity analyses were also employed for all outcomes to assess any exaggerated effects of a single study on the pooled effect size. To assess the possibility of publication bias, funnel plots were constructed using STATA 13.1, and the Egger’s test was also performed. Statistical results were deemed statistically significant if *p* < 0.05.

## 3. Results

**Figure 1:**
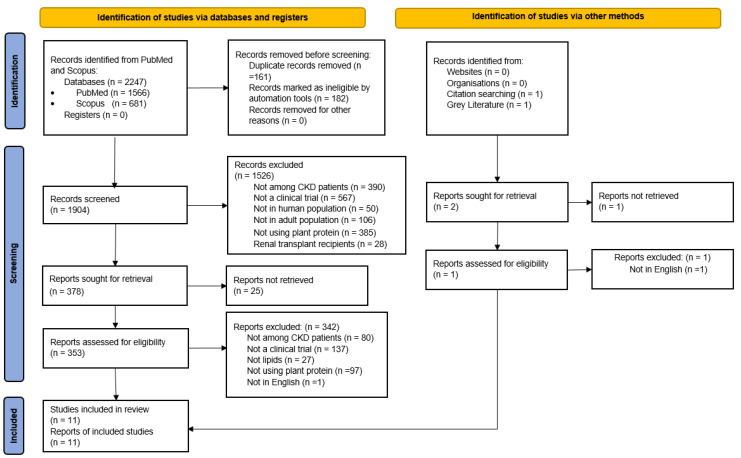
PRISMA flowchart outlining the selection process.

The study selection process is illustrated in Figure 1. A comprehensive search of PubMed, Scopus, and gray literature sources yielded 2249 study reports. After screening titles and abstracts and removing duplicates, 353 full-text reports were assessed for eligibility. Eleven reports of controlled trials, encompassing a total of 248 patients, met the inclusion criteria and were included in this meta-analysis.

The main characteristics of the eligible studies are detailed in Table 1.

Five studies [24,25,28,29,30] are randomized controlled crossover trials, while one study [31] is a non-randomized crossover controlled trial. The remaining studies [22,23,32,33,34] are randomized controlled parallel trials.

Only two of the eleven studies employed blinding for both the participants and the researchers [22,23]. Out of a total of 248 patients, 99 were end-stage kidney disease patients requiring renal replacement therapy.

**Table 1:** Characteristics of controlled clinical trials that assessed the impact of increased plant protein intake on the lipid profile of adult CKD patients.

Author, Year	Country	Study Type	Number of Study Participants	RRT	Intervention	Control	Duration (Weeks)
Ahmed, 2011 [32]	Brazil	RP	18	None	100% vegetable protein	100% animal protein	8
Anderson, 1998 [28]	USA	RC	8	None	50% soy protein	50% animal protein	8
Azadbakht, 2003 [29]	Iran	RC	14	None	35% soy + 30% vegetable protein	70% animal protein	7
Azadbakht, 2008 [33]	Iran	RP	41	None	35% soy + 30% vegetable protein	70% animal protein	192
Chen, 2005 [22]	Taiwan	RP	37	Hemodialysis	30 g soy protein/d	30 g cow milk protein/d	12
Chen, 2006 [23]	Taiwan	RP	26	Hemodialysis	30 g soy protein/d	30 g milk protein/d	12
D’Amico, 1992 [31]	Italy	NRC	20	None	100% vegetable protein	Usual CKD diet ^1^	8
Miraghajani, 2013 [24]	Iran	RC	25	None	240 mL soy milk/d	240 mL cow milk/d	4
Soroka, 1998 [25]	Israel	RC	9	None	>50% soy protein	>50% animal protein	24
Tabibi, 2010 [34]	Iran	RP	36	Peritoneal dialysis	14 g soy protein/d	Usual CKD diet ^1^	8
Teixeira, 2004 [30]	USA	RC	14	None	50% soy protein	50% animal protein	8

RP: Randomized parallel controlled trial; RC: Randomized crossover controlled trial; NRC: Non-randomized crossover controlled trial; RRT: Renal replacement therapy; ^1^: The usual CKD diet is generally composed of at least 50% animal protein.

Concerning the intervention, the majority of studies used soy as the sole source of plant protein. The total amount of protein intake ranged between 0.75 and 1.2 g/kg/day and was generally consistent across the intervention and control groups, except for the study by D’Amico et al., in which the protein intake of the control group was not specified [31].

Among the eleven studies included, four [25,28,29,32] did not specify the methods used to measure lipid levels. In the remaining studies [22,23,24,30,31,33,34], total cholesterol and triglyceride levels were assessed using enzymatic reagents. Apolipoprotein A and Apolipoprotein B were measured through either immunoturbidometry [22,23] or immunonephelometry [31]. HDL levels were determined using homogenous assays [22,23,24,34] or precipitation methods [30,31,33]. Finally, LDL was quantified either through calculation using the Friedewald formula [31,33] or through direct measurement with enzymatic reagents [22,23,24,30,34].

In their analysis, Chen et al. [22] present results stratified by the patients’ lipidemic profile at baseline (hyperlipidemic vs. normolipidemic). Therefore, data for these two groups were recorded separately throughout this meta-analysis and are presented as «Chen 2005 (h)» for the hyperlipidemic group and «Chen 2005 (n)» for the normolipidemic group.

### 3.1. Risk of Bias Assessment

The risk of bias assessment is presented in Supplementary Tables S1 and S2. Using the Cochrane RoB 2 and the ROBINS-I assessment tool, four studies [24,31,32,34] (Ahmed 2011, D’Amico 1992, Miraghajani 2013, and Tabibi 2010) were judged to be at high risk of bias, primarily due to insufficient or inconsistent data regarding the randomization process, failure to apply an intention-to-treat analysis, or missing outcome data. The remaining seven studies [22,23,25,28,29,30,33] were deemed to have some concerns regarding possible risk of bias.

### 3.2. Outcomes

#### 3.2.1. Total Cholesterol

**Figure 2:**
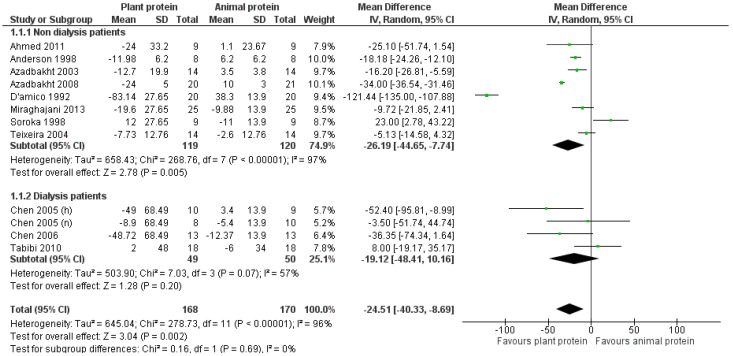
Meta-analysis of controlled trials assessing the effect of increased plant protein intake on total cholesterol levels of non-dialysis and dialysis CKD patients [22,23,24,25,28,29,30,31,32,33,34].

Overall, compared to the consumption of the usually recommended CKD diet, in which the major sources of protein are of animal origin, the increased intake of plant protein led to a statistically significant reduction of total cholesterol by 24.5 mg/dL [pooled MD = −24.51 mg/dL favoring the plant protein group, (95% CI −40.33, −8.69), *I*^2^ = 96%] (Figure 2).

In studies that include only non-dialysis CKD patients [24,25,28,29,30,31,32,33], increased plant protein intake resulted in a statistically significant reduction of total cholesterol [pooled MD = −26.19 mg/dL favoring the plant protein group, (95% CI −44.65, −7.74), *I*^2^ = 97%] (Figure 2). In studies that included dialysis patients [22,23,34], total cholesterol decreased in the intervention group, but, compared to the control group, this reduction was not statistically significant [pooled MD = −19.12 mg/dL (95% CI −48.41, 10.16), *I*^2^ = 57%] (Figure 2).

**Figure 3:**
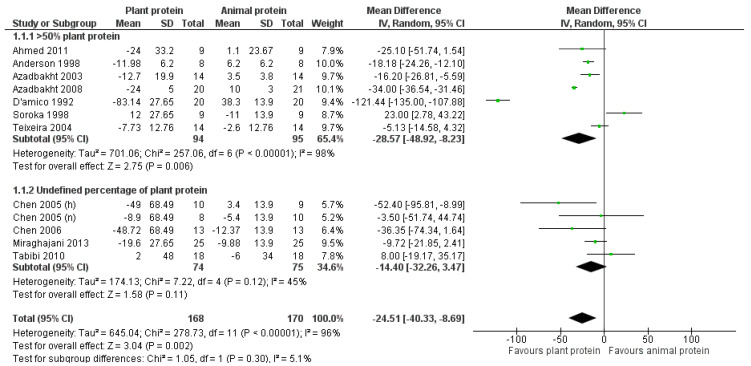
Meta-analysis of controlled trials assessing the effect of increased plant protein intake on total cholesterol levels of CKD patients according to the percentage of plant protein compared to total protein administered [22,23,24,25,28,29,30,31,32,33,34].

Among the studies in which plant protein constituted at least 50% of the total protein intake, a significant reduction in total cholesterol was observed [pooled MD = −28.57 mg/dL favoring the plant protein group, (95% CI −48.92, −8.23), *I*^2^ = 98%] (Figure 3). Regarding the studies in which the percentage increase in plant protein was not defined [22,23,24,34], the intervention led to a reduction in total cholesterol, although it was not statistically significant [pooled MD = −14.4 mg/dL (95% CI −32.26, 3.47), *I*^2^ = 45%] (Figure 3).

Following a subgroup analysis of the included studies based on their design (parallel or crossover), a statistically significant reduction in total cholesterol was observed in studies with a parallel design [22,23,32,33,34], in favor of the increased consumption of plant protein [pooled MD = −24.54 mg/dL (95% CI −40.84, −8.24), *I*^2^ = 57%] (Figure S1). Among the crossover studies [24,25,28,29,30,31], the reduction in total cholesterol was not statistically significant [pooled MD = −24.8 mg/dL (95% CI −54.91, 5.31), *I*^2^ = 98%] (Figure S1).

After excluding the four studies with high Rob2 scores [24,31,32,34], or the five studies [22,23,24,25,31] with imputed standard deviations, the beneficial effect of increased plant protein consumption remained; however, the reduction in total cholesterol was smaller [pooled MD = −15.92 mg/dL (95% CI −28.88, −2.96), *I*^2^ = 92% and pooled MD = −18.66 mg/dL (95% CI −30.61, −6.7), *I*^2^ = 91%, respectively] (Figures S2 and S3).

A leave-one-out sensitivity analysis was also carried out, which indicated the impact of the outlying study by D’Amico et al. [31] on the pooled result. After the exclusion of this study, the reduction in total cholesterol levels associated with higher plant protein intake was attenuated but remained statistically significant [pooled MD = −14.11 mg/dL (95% CI −25.20, −3.02), *I*^2^ = 90%].

#### 3.2.2. Triglycerides

**Figure 4:**
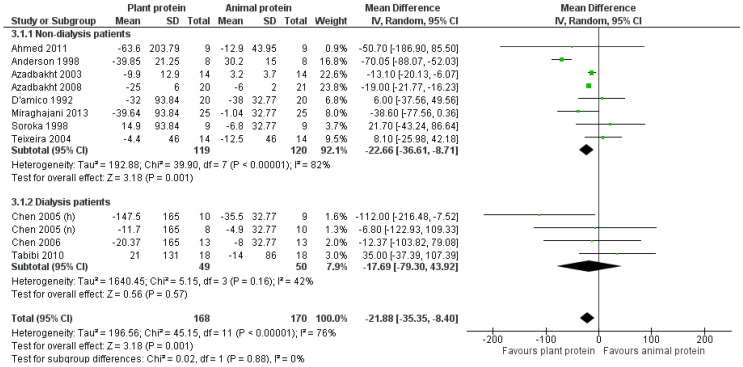
Meta-analysis of controlled trials assessing the effect of increased plant protein intake on triglyceride levels of non-dialysis and dialysis CKD patients [22,23,24,25,28,29,30,31,32,33,34].

The overall pooled result of the studies indicated a statistically significant reduction in triglycerides of 21.9 mg/dL following increased consumption of plant protein compared to the usually prescribed CKD diet [pooled MD = −21.88 mg/dL (95% CI −35.35, −8.4), *I*^2^ = 76%] (Figure 4). This primary result was further supported within the non-dialysis population [24,25,28,29,30,31,32,33], with the reduction being almost identical [pooled MD = −22.66 mg/dL (95% CI −36.61, −8.71), *I*^2^ = 82%] (Figure 4). However, the reduction of triglycerides was not statistically significant in the dialysis population [22,23,34] [pooled MD = −17.69 mg/dL (95% CI −79.3, 43.92), *I*^2^ = 42%] (Figure 4).

**Figure 5:**
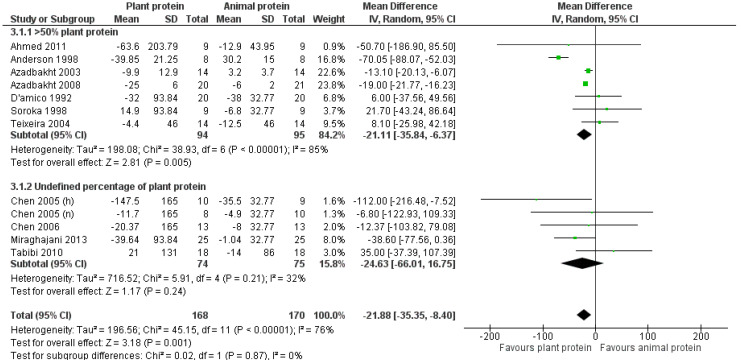
Meta-analysis of controlled trials assessing the effect of increased plant protein intake on triglyceride levels of CKD patients according to the percentage of plant protein compared to total protein administered [22,23,24,25,28,29,30,31,32,33,34].

Upon isolating the seven studies in which the percentage of plant protein constituted at least 50% of the total protein intake [25,28,29,30,31,32,33], a statistically significant reduction in triglycerides was observed in favor of the intervention [pooled MD = −21.11 mg/dL (95% CI −35.84, −6.37), *I*^2^ = 85%] (Figure 5).

Concerning the effect of the study design on the pooled result, a reduction in triglycerides was observed in the subgroup of studies with a parallel design [22,23,32,33,34] [pooled MD = −18.54 mg/dL (95% CI −37.42, 0.34), *I*^2^ = 8%] and among those with a crossover design [24,25,28,29,30,31] [pooled MD = −18.68 mg/dL (95% CI −47.59, 10.23), *I*^2^ = 87%]; however, it was not statistically significant (Figure S4).

Our primary result concerning triglycerides was upheld following the exclusion of studies with a high risk of bias [24,31,32,34] [pooled MD = −24.35 mg/dL (95% CI −39.53, −9.18), *I*^2^ = 83%] or with imputed standard deviations [22,23,24,25,31] [pooled MD = −25.13 mg/dL (95% CI −40.69, −9.31), *I*^2^ = 85%] (Figures S5 and S6).

Notably, in the analysis of how plant protein intake affects triglycerides levels among CKD patients, the leave-one-out method did not reveal any study that was particularly influential to the overall outcome.

#### 3.2.3. LDL Cholesterol

**Figure 6:**
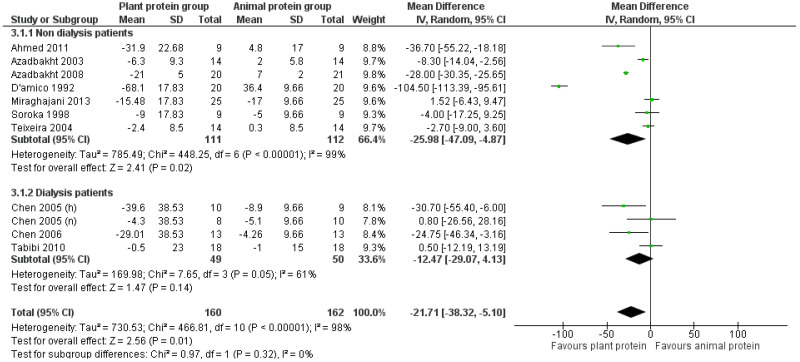
Meta-analysis of controlled trials assessing the effect of increased plant protein intake on LDL cholesterol levels of non-dialysis and dialysis CKD patients [22,23,24,25,29,30,31,32,33,34].

Following the increased consumption of plant protein in CKD patients, the overall pooled analysis of the eligible studies indicated a statistically significant reduction in LDL cholesterol by 21.7 mg/dL [pooled MD = −21.71 mg/dL (95% CI −38.32, −5.1), *I*^2^ = 98%]. The beneficial effect of increased plant protein intake was observed in all CKD patients, although it was less pronounced among patients receiving dialysis [pooled MD = −12.47 mg/dL (95% CI −29.07, −4.13), *I*^2^ = 61%] (Figure 6).

**Figure 7:**
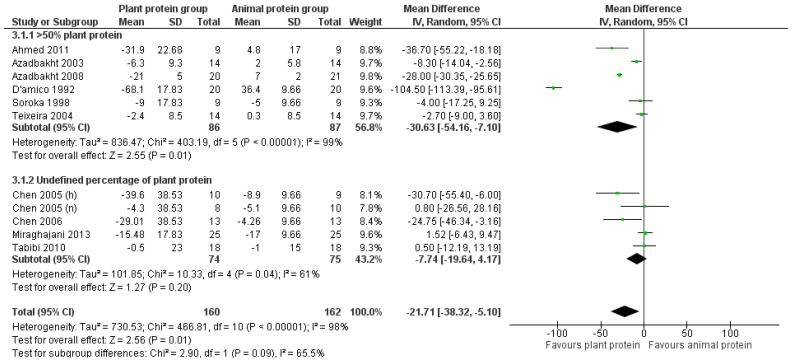
Meta-analysis of controlled trials assessing the effect of increased plant protein intake on LDL cholesterol levels of CKD patients according to the percentage of plant protein compared to total protein administered [22,23,24,25,29,30,31,32,33,34].

Upon isolating the studies in which the percentage of plant protein intake was at least 50% [25,29,30,31,32,33], a greater reduction in LDL was observed [pooled MD = −30.63 mg/dL, in favor of the intervention (95% CI −54.17, −7.10), *I*^2^ = 99%] (Figure 7).

The statistically significant reduction in LDL cholesterol levels remained after the exclusion of studies with a high risk of bias, although it was less prominent [24,31,32,34] [pooled MD = −13.65 mg/dL (95% CI −25.8, −1.5), *I*^2^ = 94%] or with imputed standard deviations [22,23,24,25,31] [pooled MD = −16.25 mg/dL (95% CI −29.27, −3.23), *I*^2^ = 95%] (Figures S7 and S8).

Two [31,33] of the three studies [30,31,33] that used the precipitation method to assess HDL levels applied the Friedewald formula to calculate LDL cholesterol levels. To determine whether this indirect method for LDL measurement could have introduced bias, we repeated the analysis after excluding these two studies. The beneficial effect was attenuated but remained statistically significant [pooled MD = −8.4 mg/dL (95% CI −15.18, −1.63), *I*^2^ = 66%].

Following a leave-one-out sensitivity analysis, the impact of the D’Amico et al. [31] study contributing an exaggerated pooled mean difference was confirmed. The meta-analysis was repeated after excluding this study, which resulted in an attenuated but statistically significant reduction in LDL cholesterol levels [pooled MD = −12.49 mg/dL (95% CI −23.04, −1.93), *I*^2^ = 94%].

#### 3.2.4. HDL Cholesterol

**Figure 8:**
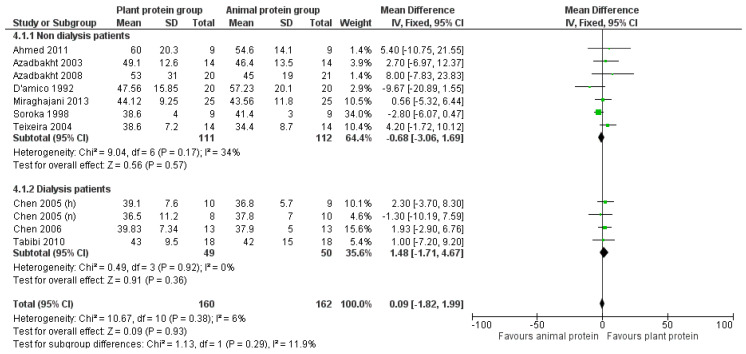
Meta-analysis of controlled trials assessing the effect of increased plant protein intake on HDL cholesterol levels of non-dialysis and dialysis CKD patients [22,23,24,25,29,30,31,32,33,34].

**Figure 9:**
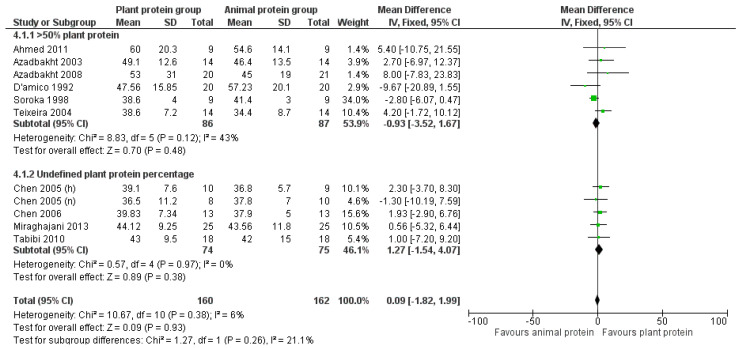
Meta-analysis of controlled trials assessing the effect of increased plant protein intake on HDL cholesterol levels of CKD patients according to the percentage of plant protein compared to total protein administered [22,23,24,25,29,30,31,32,33,34].

Overall, no biologically relevant or statistically significant association was found between increased plant protein intake and HDL cholesterol levels [pooled MD = 0.09 mg/dL (95% CI −1.82, 1.99), *I*^2^ = 6%] (Figure 8). No change in this outcome was observed after conducting sensitivity analyses based on CKD stage or percentage of plant protein intake (Figure 8 and Figure 9).

Similarly, after excluding the four studies that were judged to have a high risk of bias [24,31,32,34] or the three studies [30,31,33] that used the precipitation method to estimate HDL cholesterol, no significant association was found [pooled MD = 0.22 mg/dL (95% CI −1.91, 2.35), *I*^2^ = 17% and pooled MD = −0.22 mg/dL, favoring the control group (95% CI −2.28, 1.84), *I*^2^ = 0% respectively] (Figure S9).

Lastly, no study appeared to have an impact on HDL levels after repeating the analysis, excluding one study each time (leave-one-out method).

#### 3.2.5. Apolipoprotein A

**Figure 10:**
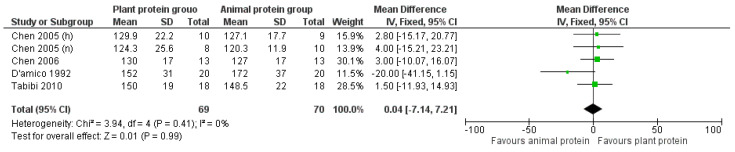
Meta-analysis of controlled trials assessing the effect of increased plant protein intake on Apolipoprotein A levels of CKD patients [22,23,31,34].

Overall, following the increased consumption of plant protein, no significant differences in Apolipoprotein A levels were observed [pooled MD = 0.04 mg/dL (95% CI −7.14, 7.21), *I*^2^ = 0%] (Figure 10). However, when the analysis was repeated after excluding the outlying D’Amico et al. [31] study, the Apo A levels of CKD patients increased by 2.64 mg/dL; however, this finding was not statistically significant [pooled MD = 2.64 mg/dL (95% CI −4.99, 10.26), *I*^2^ = 0%].

#### 3.2.6. Apolipoprotein B

**Figure 11:**
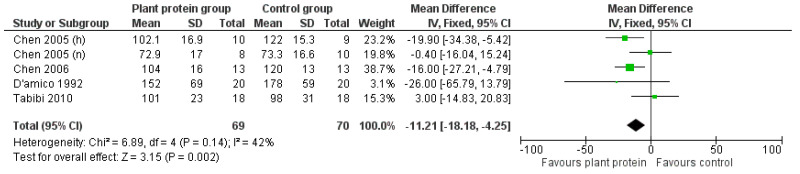
Meta-analysis of controlled trials assessing the effect of increased plant protein intake on Apolipoprotein B levels of CKD patients [22,23,31,34].

Finally, a beneficial impact was found on Apolipoprotein B levels, which were shown to be decreased by 11.21 mg/dL following the increased consumption of plant protein [pooled MD = −11.21 mg/dL (95% CI −18.18, −4.25), *I*^2^ = 42%] (Figure 11).

## 4. Publication Bias

We assessed the possibility of publication bias through visual inspection of funnel plots (Figures S10–S13) and the Egger regression test [35]. In our analysis, although Egger’s regression test did not indicate publication bias across any of our outcomes, the funnel plots appeared asymmetrical. Specifically, for HDL and triglycerides, smaller, less precise studies were asymmetrically distributed (Figures S11 and S13), while for total cholesterol and LDL, the distribution of studies was diffusely asymmetrical (Figures S10 and S12). While the high degree of heterogeneity in total cholesterol and LDL outcomes may have contributed to the observed asymmetry, the presence of publication bias cannot be ruled out.

## 5. Discussion

Our meta-analysis of clinical trials assessing the effect of plant protein intake on the lipidemic profile of adult CKD patients demonstrates that increased plant protein intake, compared to an animal-based diet, resulted in a statistically significant reduction of 24 mg/dL in total cholesterol, 22 mg/dL in LDL cholesterol, 22 mg/dL in triglycerides, and 11 mg/dL in Apolipoprotein B levels. No significant associations were found between increased plant protein intake and HDL cholesterol or Apolipoprotein A levels.

Our findings align with those of Zhang et al. [18], who conducted a meta-analysis of nine studies on the impact of soy protein on lipid levels in pre-dialysis CKD patients. Although they observed reductions in triglycerides and total cholesterol, these changes were not statistically significant.

In the general population, Li et al. [36] conducted a thorough meta-analysis of 112 trials involving 5582 participants, which indicated that increased plant protein intake could decrease LDL cholesterol by 6 mg/dL (95% CI −7, −4) and Apolipoprotein B by 5 mg/dL (95% CI −6, −3).

The more pronounced reductions in total cholesterol and LDL cholesterol observed in our analysis—in comparison to the existing literature—could be partially attributed to the study by D’Amico et al. [31], which was shown to produce an exaggerated pooled effect estimate. Following its exclusion, the reductions in total cholesterol and LDL cholesterol associated with higher plant protein intake were 14.11 mg/dL (95% CI −25.20, −3.02) and 12.49 mg/dL (95% CI −23.04, −1.93), respectively.

Remarkably, despite the beneficial effects of plant protein on most lipid parameters, the majority of the existing literature—including our findings—has observed little to no impact on HDL cholesterol or Apolipoprotein A levels. Meta-analyses in the general population have reported varying effects of plant protein on HDL cholesterol, with some showing a modest increase of up to 2.7 mg/dL [37,38,39,40] and others noting a slight decrease [41,42,43]. Similarly, the effect of plant protein on Apolipoprotein A levels appears to follow this variable pattern [44,45,46,47,48].

Appropriate lipid regulation is crucial for CKD patients, not only to mitigate the risk of cardiovascular disease but also to address the nephrotoxic effects of dyslipidemia. Elevated circulating levels of LDL cholesterol are linked to mesangial cell proliferation, increased chemokine production, and macrophage recruitment. This cascade of events can lead to focal segmental glomerulosclerosis through mechanisms similar to those of atherosclerosis [49,50,51]. Additionally, hypertriglyceridemia has been shown to exacerbate podocyte injury, further contributing to glomerulosclerosis [50,52]. Recent cohort studies have underscored the relationship between lipid regulation and the improvement of kidney disease progression [53,54,55]. Specifically, three large cohort studies conducted in Korea and Italy have demonstrated that hypertriglyceridemia and elevated levels of Apolipoprotein B and LDL cholesterol are independently associated with a decline in the glomerular filtration rate (GFR) and the progression to end-stage renal disease [53,54,55].

Soy was exclusively used as a source of plant protein in varying amounts for the majority of the included studies. Azadbakht et al. [29,33] applied a diet consisting of 35% soy protein and 30% non-soy vegetable protein, but the exact sources were not specified. Thus, although our findings likely reflect the effect of increased intake of soy protein rather than other plant protein sources, the inclusion of the Azadbakht et al. studies [29,33] limits the generalizability of our results to soy protein alone.

It is noted that the safety profile and benefits of soy protein have been well researched within the renal disease population, presumably due to the prolonged lifespan of these patients in East Asian countries, where there is a tradition of consuming soy products [56,57,58]. The main safety concerns lie in the risk of developing hyperphosphatemia and hyperkalemia; however, these concerns are not supported by current evidence. In two meta-analyses of pre-dialysis CKD patients, soy intake was associated with lower serum phosphorus levels, while a clinical trial involving dialysis patients—who are at the highest risk of developing hyperkalemia—failed to find an effect of soy consumption on potassium levels in the blood [18,59,60]. These meta-analyses also highlight the potential renoprotective effects of soy protein, showing small but statistically significant reductions in serum creatinine [18,59] and proteinuria [59]. Moreover, animal studies have provided insight into the counteractive pathways of soy protein regarding oxidative stress and fibrosis, two important prognostic factors in CKD progression [61,62,63,64]. As an example, in mouse models of kidney disease, soy protein appears to increase antioxidant capacity by increasing catalase and decreasing lipid peroxidation [65], as well as improving the histopathological findings of fibrosis, such as mesangial matrix expansion, tubulointerstitial fibrosis, and myofibroblast differentiation [66].

When considering the generalizability of our results, certain limitations need to be taken into account. First, the relatively small number of studies included in some analyses renders the pooled estimates susceptible to type 1 error [67,68]. Of the eleven studies included, four were judged to be at a high risk of bias [24,31,32,34], mainly due to an inadequate randomization process, missing outcome data, and failure to apply an intention-to-treat analysis.

Another limitation of our study lies in the high degree of heterogeneity observed for the pooled estimates of total cholesterol (*I*^2^ = 97%) and LDL cholesterol (*I*^2^ = 98%). Heterogeneity persisted even after conducting subgroup analyses based on CKD stage, percentage of plant protein, study design, risk of bias, and the imputation of missing standard deviations. A small reduction in the degree of heterogeneity (90% and 94%, respectively) was observed after excluding the study by D’Amico et al. [31], which presented exaggerated effect estimates for these values. This study is a non-randomized crossover trial in which, following a baseline free diet, participants were placed on a strict vegetarian diet for 8 weeks, with both protein and fatty acid sources replaced by vegetarian alternatives. It is therefore likely that the amplified reductions in total cholesterol and LDL cholesterol observed in this trial are attributable to differences in total calorie intake and fatty acid composition between the two diets, rather than the effect of plant protein alone.

Incomplete data limited the thorough exploration of heterogeneity. For example, a possible effect modifier of the impact of plant protein on lipid levels is the baseline lipid status. Higher baseline values may potentially amplify the impact of plant protein [69,70]. However, within our sample, only one study [22] stratified results according to baseline lipid status, preventing a comprehensive assessment of this factor’s contribution to heterogeneity. Similarly, the exploration of heterogeneity was limited by insufficient information on the use of lipid-lowering agents in the majority of the included studies. Nevertheless, lipid-lowering drug use could introduce bias into our analysis only if there were significant differences in their administration between the intervention and control groups. Considering that all of our studies, except one (D’amico et al. [31] in which patients receiving lipid-lowering agents were excluded), are randomized controlled studies, it is unlikely that such differences would exist. In addition, the methods used for lipid measurement can also be a source of heterogeneity. However, it was not possible to explore this further, as four out of the eleven studies considered in this analysis did not report on the methods used.

Another possible source of heterogeneity is the quality of plant protein, which is related to the different forms of industrial processing and their impact on the protein’s digestibility throughout the intestinal tract. Soy protein exhibits broad variations in DIAAS (Digestible Indispensable Amino Acid Score), a measure of digestibility [71,72]. Given the wide time span over which our studies were published, it is plausible that changes in processing methods over time could have contributed to variations in protein quality, further influencing heterogeneity.

A more comprehensive assessment of heterogeneity is additionally limited by the incomplete exploration of the underlying pathophysiological pathways. A deeper understanding of these pathways could clarify how specific properties of plant proteins interact with blood lipids, enabling targeted research and potentially more informative results.

Another limitation of our study concerns the short duration of the interventions, which does not allow us to draw direct conclusions about the effect of increased plant protein intake on hard outcomes such as major cardiovascular events or death. Short duration is often acknowledged as a limitation of clinical trials, which often aim to elucidate the impact of different therapies/interventions on biomarkers of effect rather than on the disease itself. In this context, blood lipids serve as a reliable biomarker of effect, as the association between lipid regulation and cardiovascular risk has been well established in the medical literature [73,74].

Our analysis has several strengths. The eligible studies were all controlled clinical trials, retrieved through an extensively thorough search of up-to-date databases and gray literature sources. In comparison to the existing literature, our study contributes by providing pooled estimates concerning the effect of increased plant protein intake on LDL, HDL, ApoA, and ApoB levels (while in previous analyses, effect estimates were limited to TC and TG). CKD patients of all stages were also included, enabling a global application of findings to this population and revealing gaps in the existing literature.

## 6. Conclusions

In conclusion, we found evidence that increased plant protein intake (primarily achieved through increases in soy protein consumption) can reduce total cholesterol, LDL cholesterol, triglycerides, and Apolipoprotein B levels in adult CKD patients. However, further research is required to explore the effects of plant protein intake on lipid levels within this population. Long-term randomized studies with larger samples and an emphasis on dialysis populations in which hard outcomes (such as major cardiovascular events or death) could also be accounted for, could help clarify the existing evidence. In addition, there is a significant literature gap concerning the effect (and safety profile) of non-soy plant protein sources on blood lipids, as well as the effect of plant protein on important blood lipid indicators such as VLDL, Non-HDL, ApoA, or ApoB.

## Data Availability

Due to privacy reasons, the data presented in this study are available upon request from the corresponding author.

## Supplementary Materials

The following supporting information can be downloaded at https://www.mdpi.com/article/10.3390/nu17091408/s1: Table S1. Summary of risk of bias assessment of randomized controlled trials using the ROB-2 score; Table S2. Risk of bias assessment for the D’Amico et al. [31] study using the ROBINS-I score; Table S3. Search terms and Boolean operators used for each database; Figure S1. Meta-analysis assessing the effect of increased plant protein intake on total cholesterol levels of CKD patients according to study design; Figure S2. Meta-analysis assessing the effect of increased plant protein intake on total cholesterol levels of CKD patients according to risk of bias; Figure S3. Meta-analysis assessing the effect of increased plant protein intake on total cholesterol levels of CKD patients according to the imputation of standard deviations; Figure S4. Meta-analysis assessing the effect of increased plant protein intake on triglyceride levels of CKD patients according to study design; Figure S5. Meta-analysis assessing the effect of increased plant protein intake on triglyceride levels of CKD patients according to risk of bias; Figure S6. Meta-analysis assessing the effect of increased plant protein intake on triglyceride levels of CKD patients according to the imputation of standard deviations; Figure S7. Meta-analysis assessing the effect of increased plant protein intake on LDL cholesterol levels of CKD patients according to risk of bias; Figure S8. Meta-analysis assessing the effect of increased plant protein intake on LDL cholesterol levels of CKD patients according to the imputation of standard deviations; Figure S9. Meta-analysis assessing the effect of increased plant protein intake on HDL cholesterol levels of CKD patients according to risk of bias; Figure S10. Funnel plots of the standard errors (SE) versus the mean differences (MD) of total cholesterol levels; Figure S11. Funnel plots of SE versus MD of triglycerides levels; Figure S12. Funnel plot of SE versus MD of LDL cholesterol levels; Figure S13. Funnel plot of SE versus MD of HDL cholesterol levels.
