# Supplementary material for: The Effect of Increased Plant Protein Intake on the Lipid Profile of Chronic Kidney Disease Patients: A Meta-Analysis of Controlled Clinical Trials
Source: Nutrients. 2025 Apr 23;17(9):1408. doi: 10.3390/nu17091408 (PMC12073598; doi:10.3390/nu17091408)
Supplement: Supplementary file 1 [file nutrients-17-01408-s001.zip › nutrients-3524916-supplementary.pdf]

Table S1. Summary of risk of bias assessment of randomized controlled trials using the ROB-2 score.

	Teixeira 2004	Tabibi 2010	Soroka 1998	Miraghajani 2013	Chen 2006	Chen 2005 (n)	Chen 2005 (h)	Azadbakht 2008	Azadbakht 2003	Anderson 1998	Ahmed 2011
Bias arising from the randomization process	?	−	?	−	?	?	?	?	?	?	−
Bias due to deviations from the intended interventions	+	?	?	?	?	?	?	?	+	+	−
Bias due to missing outcome data	?	+	?	+	+	+	+	+	+	+	−
Bias in the measurement of the outcome	+	+	+	+	+	+	+	+	+	+	+
Bias in the selection of the reported result	+	+	+	+	+	+	+	?	+	+	+
Total risk of bias	?	−	?	−	?	?	?	?	?	?	−

Judgment

− High

? Some concerns

\+ Low

	D'amico 1992
Bias due to confounding	?
Bias in classification of interventions	−
Bias of selection of participants	+
Bias due to deviations of intended interventions	+
Bias due to missing data	+
Bias in measurement of the outcome	+
Bias in selection of the reported result	+
Total risk of bias	−

Table S2. Risk of bias assessment for the D'Amico et al. study using the ROBINS-I score.

	PubMed	Scopus
1	((«Glomerular filtration Rate»[Mesh]) OR (GFR) OR («Glomerular Filtration Rate») OR («Kidney diseases»[Mesh]) OR («Renal failure») OR («kidney disease») OR («renal disease») OR («renal insufficiency») OR («mild to moderate kidney disease») OR (mmkd) OR («chronic kidney disease») OR («kidney failure») OR (ckd) OR («renal insufficiency»[Mesh]) OR («Proteinuria»[Mesh]) OR (albuminuri*) OR (proteinuri*) OR («renal replacement therapy»[Mesh]) OR (hemodialys*) OR (hemodiafiltrat*) OR (dialys*) OR (dialytic) OR («peritoneal dialysis»))	TITLE-ABS("kidney disease" OR "renal disease" OR "renal insufficiency" OR "renal failure" OR mmkd OR "glomerular filtration rate" OR "chronic kidney disease" OR "kidney failure" OR GFR OR albuminuria OR proteinuria OR hemodialysis OR dialysis OR hemodiafiltration OR "renal replacement therapy" OR "peritoneal dialysis") AND TITLE-ABS("plant protein" OR "soybean protein" OR "soy protein" OR soymilk OR "pea proteins" OR "nut protein" OR "dietary protein" OR "vegetable protein" OR "vegetarian protein" OR "vegan protein" OR "whey protein" OR "casein protein" OR "grain protein" OR "tofu protein" OR "tempeh protein" OR "legume protein" OR "oat protein" OR "bean protein" OR "milk protein" OR "casein protein" OR "fruits protein" OR "rice protein" OR "animal protein")
2	((«Plant proteins»[Mesh]) OR («plant protein») OR («soybean proteins»[Mesh]) OR («soy protein») OR («Soy Milk»[Mesh]) OR (soymilk) OR («pea proteins»[Mesh]) OR («pea proteins») OR («nut protein»[Mesh]) OR («nut protein») OR («dietary proteins»[Mesh]) OR («dietary proteins») OR («vegetable protein») OR («vegetarian protein») OR («vegan protein») OR («grain protein») OR («tofu protein») OR («tempeh protein») OR («legume protein») OR («oat protein») OR («bean protein») OR («fruits protein») OR («rice protein») OR («whey protein») OR («casein protein») OR («milk protein») OR («animal protein»))	(TITLE-ABS-KEY (("clinical trials" OR "clinical trials as a topic" OR "randomized controlled trial" OR "Randomized Controlled Trials as Topic" OR "controlled clinical trial" OR "Controlled Clinical Trials as Topic" OR "random allocation" OR "randomly allocated" OR "allocated randomly" OR "Double-Blind Method" OR "Single-Blind Method" OR "Cross-Over Studies" OR "Placebos" OR "cross-over trial" OR "single blind" OR "double blind" OR "factorial design" OR "factorial trial"))) OR (TITLE-ABS (clinical trial* OR trial* OR rct* OR random* OR blind*))
3	(randomized controlled trial[pt] OR controlled clinical trial[pt] OR randomized[tiab] OR placebo[tiab] OR drug therapy[sh] OR randomly[tiab] OR trial[tiab] OR groups[tiab]) NOT (animals [mh] NOT humans [mh])	(INDEXTERMS ("clinical trials" OR "clinical trials as a topic" OR "randomized controlled trial" OR "Randomized Controlled Trials as Topic" OR "controlled clinical trial" OR "Controlled Clinical Trials" OR "random allocation" OR "Double-Blind Method" OR "Single-Blind Method" OR "Cross-Over Studies" OR "Placebos" OR "multicenter study" OR "double blind procedure" OR "single blind procedure" OR "crossover procedure" OR "clinical trial" OR "controlled study" OR "randomization" OR "placebo"))
4	1 AND 2 AND 3	1 AND 2 AND 3

Table S3. Search terms and Boolean operators used for each database.

Study or Subgroup	Plant protein Mean	Plant protein SD	Plant protein Total	Animal protein Mean	Animal protein SD	Animal protein Total	Weight	Mean Difference IV, Random, 95% CI
**1.1.1 Crossover studies**								
Anderson 1998 [28]	-11.98	6.2	8	6.2	6.2	8	10.0%	-18.18 [-24.26, -12.10]
Azadbakht 2003 [29]	-12.7	19.9	14	3.5	3.8	14	9.7%	-16.20 [-26.81, -5.59]
D'amico 1992 [31]	-83.14	27.65	20	38.3	13.9	20	9.4%	-121.44 [-135.00, -107.88]
Miraghajani 2013 [24]	-19.6	27.65	25	-9.88	13.9	25	9.5%	-9.72 [-21.85, 2.41]
Soroka 1998 [25]	12	27.65	9	-11	13.9	9	8.7%	23.00 [2.78, 43.22]
Teixeira 2004 [30]	-7.73	12.76	14	-2.6	12.76	14	9.7%	-5.13 [-14.58, 4.32]
**Subtotal (95% CI)**			**90**			**90**	**57.0%**	**-24.80 [-54.91, 5.31]**
Heterogeneity: Tau² = 1374.02; Chi² = 244.36, df = 5 (P < 0.00001); I² = 98%								
Test for overall effect: Z = 1.61 (P = 0.11)								
**1.1.2 Parallel studies**								
Ahmed 2011 [32]	-24	33.2	9	1.1	23.67	9	7.9%	-25.10 [-51.74, 1.54]
Azadbakht 2008 [33]	-24	5	20	10	3	21	10.1%	-34.00 [-36.54, -31.46]
Chen 2005 (h) [22]	-49	68.49	10	3.4	13.9	9	5.7%	-52.40 [-95.81, -8.99]
Chen 2005 (n) [22]	-8.9	68.49	8	-5.4	13.9	10	5.2%	-3.50 [-51.74, 44.74]
Chen 2006 [23]	-48.72	68.49	13	-12.37	13.9	13	6.4%	-36.35 [-74.34, 1.64]
Tabibi 2010 [34]	2	48	18	-6	34	18	7.8%	8.00 [-19.17, 35.17]
**Subtotal (95% CI)**			**78**			**80**	**43.0%**	**-24.54 [-40.84, -8.24]**
Heterogeneity: Tau² = 201.64; Chi² = 11.72, df = 5 (P = 0.04); I² = 57%								
Test for overall effect: Z = 2.95 (P = 0.003)								
**Total (95% CI)**			**168**			**170**	**100.0%**	**-24.51 [-40.33, -8.69]**
Heterogeneity: Tau² = 645.04; Chi² = 278.73, df = 11 (P < 0.00001); I² = 96%								
Test for overall effect: Z = 3.04 (P = 0.002)								
Test for subgroup differences: Chi² = 0.00, df = 1 (P = 0.99), I² = 0%								

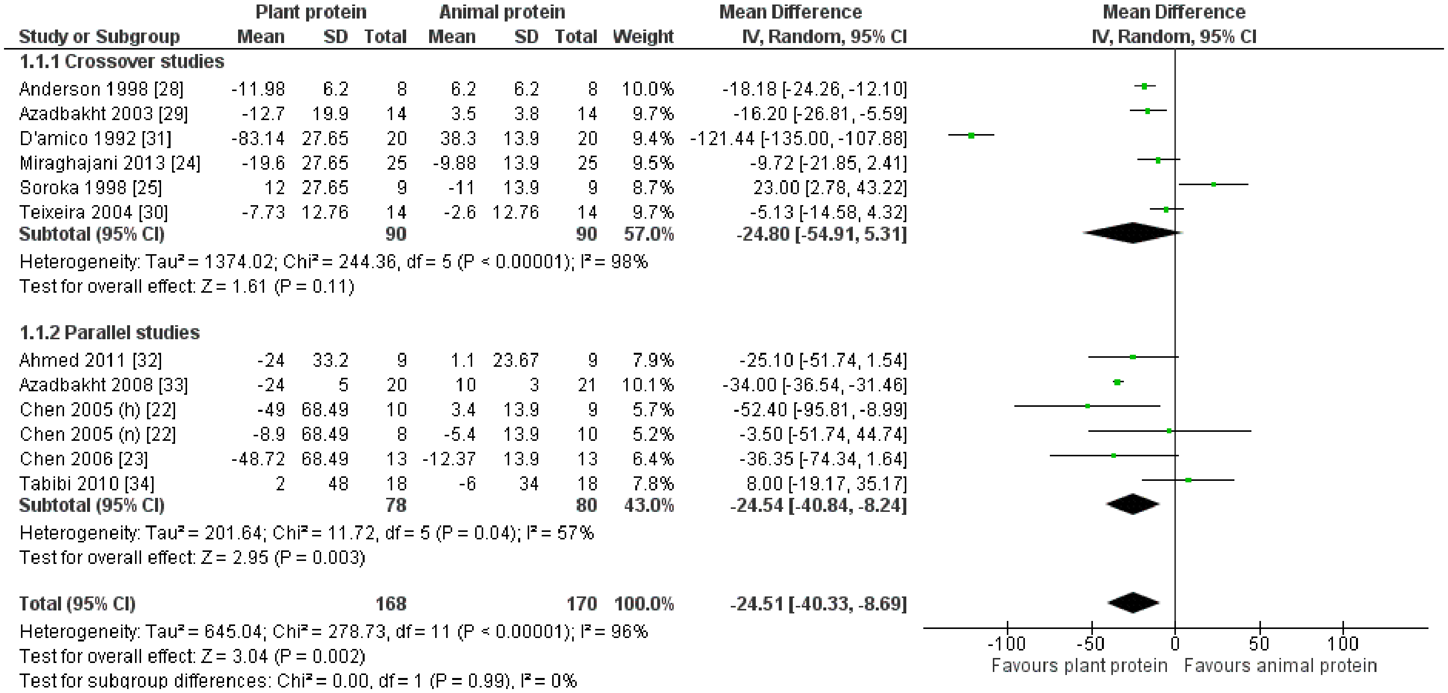

Figure S1. Meta-analysis of controlled clinical trials assessing the effect of increased plant protein intake on total cholesterol levels of CKD patients according to study design.

Study or Subgroup	Plant protein Mean	SD	Total	Animal protein Mean	SD	Total	Weight	Mean Difference IV, Random, 95% CI
**1.1.1 Some concerns about bias**								
Anderson 1998 [28]	-11.98	6.2	8	6.2	6.2	8	10.0%	-18.18 [-24.26, -12.10]
Azadbakht 2003 [29]	-12.7	19.9	14	3.5	3.8	14	9.7%	-16.20 [-26.81, -5.59]
Azadbakht 2008 [33]	-24	5	20	10	3	21	10.1%	-34.00 [-36.54, -31.46]
Chen 2005 (h) [22]	-49	68.49	10	3.4	13.9	9	5.7%	-52.40 [-95.81, -8.99]
Chen 2005 (n) [22]	-8.9	68.49	8	-5.4	13.9	10	5.2%	-3.50 [-51.74, 44.74]
Chen 2006 [23]	-48.72	68.49	13	-12.37	13.9	13	6.4%	-36.35 [-74.34, 1.64]
Soroka 1998 [25]	12	27.65	9	-11	13.9	9	8.7%	23.00 [2.78, 43.22]
Teixeira 2004 [30]	-7.73	12.76	14	-2.6	12.76	14	9.7%	-5.13 [-14.58, 4.32]
**Subtotal (95% CI)**			**96**			**98**	**65.4%**	**-15.92 [-28.88, -2.96]**
Heterogeneity: Tau² = 237.99; Chi² = 84.90, df = 7 (P < 0.00001); I² = 92%								
Test for overall effect: Z = 2.41 (P = 0.02)								
**1.1.2 High risk of bias**								
Ahmed 2011 [32]	-24	33.2	9	1.1	23.67	9	7.9%	-25.10 [-51.74, 1.54]
D'amico 1992 [31]	-83.14	27.65	20	38.3	13.9	20	9.4%	-121.44 [-135.00, -107.88]
Miraghajani 2013 [24]	-19.6	27.65	25	-9.88	13.9	25	9.5%	-9.72 [-21.85, 2.41]
Tabibi 2010 [34]	2	48	18	-6	34	18	7.8%	8.00 [-19.17, 35.17]
**Subtotal (95% CI)**			**72**			**72**	**34.6%**	**-37.49 [-103.92, 28.94]**
Heterogeneity: Tau² = 4480.72; Chi² = 169.79, df = 3 (P < 0.00001); I² = 98%								
Test for overall effect: Z = 1.11 (P = 0.27)								
**Total (95% CI)**			**168**			**170**	**100.0%**	**-24.51 [-40.33, -8.69]**
Heterogeneity: Tau² = 645.04; Chi² = 278.73, df = 11 (P < 0.00001); I² = 96%								
Test for overall effect: Z = 3.04 (P = 0.002)								
Test for subgroup differences: Chi² = 0.39, df = 1 (P = 0.53), I² = 0%								

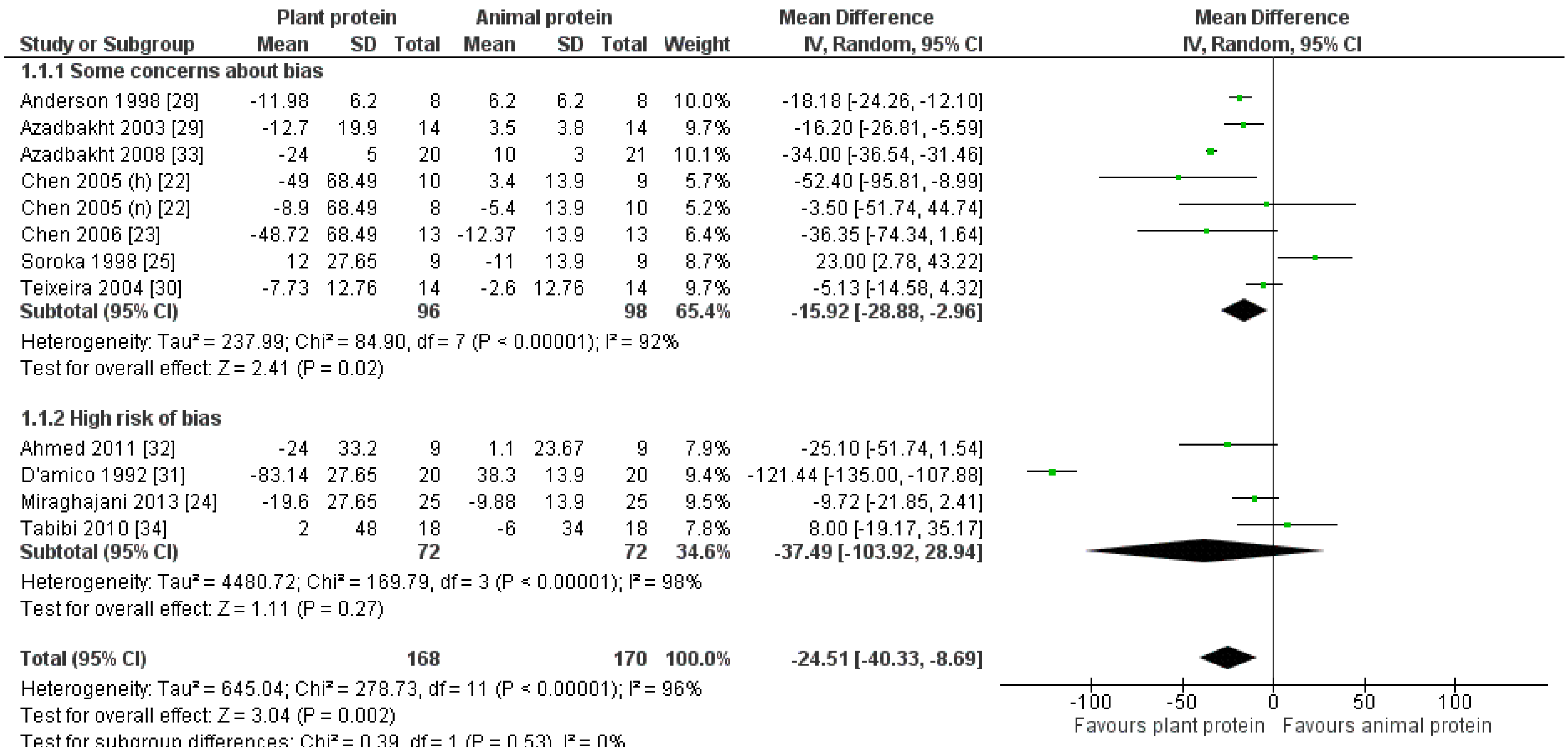

Figure S2. Meta-analysis of controlled trials assessing the effect of increased plant protein intake on total cholesterol levels of CKD patients according to risk of bias.

Study or Subgroup	Plant protein Mean	SD	Total	Animal protein Mean	SD	Total	Weight	Mean Difference IV, Random, 95% CI
**1.1.1 SD not imputed**								
Ahmed 2011 [32]	-24	33.2	9	1.1	23.67	9	7.9%	-25.10 [-51.74, 1.54]
Anderson 1998 [28]	-11.98	6.2	8	6.2	6.2	8	10.0%	-18.18 [-24.26, -12.10]
Azadbakht 2003 [29]	-12.7	19.9	14	3.5	3.8	14	9.7%	-16.20 [-26.81, -5.59]
Azadbakht 2008 [33]	-24	5	20	10	3	21	10.1%	-34.00 [-36.54, -31.46]
Chen 2005 (h) [22]	-49	68.49	10	3.4	13.9	9	5.7%	-52.40 [-95.81, -8.99]
Tabibi 2010 [34]	2	48	18	-6	34	18	7.8%	8.00 [-19.17, 35.17]
Teixeira 2004 [30]	-7.73	12.76	14	-2.6	12.76	14	9.7%	-5.13 [-14.58, 4.32]
**Subtotal (95% CI)**			**93**			**93**	**60.8%**	**-18.66 [-30.61, -6.70]**
Heterogeneity: Tau² = 184.27; Chi² = 65.42, df = 6 (P < 0.00001); I² = 91%								
Test for overall effect: Z = 3.06 (P = 0.002)								
**1.1.2 SD imputed**								
Chen 2005 (n) [22]	-8.9	68.49	8	-5.4	13.9	10	5.2%	-3.50 [-51.74, 44.74]
Chen 2006 [23]	-48.72	68.49	13	-12.37	13.9	13	6.4%	-36.35 [-74.34, 1.64]
D'amico 1992 [31]	-83.14	27.65	20	38.3	13.9	20	9.4%	-121.44 [-135.00, -107.88]
Miraghajani 2013 [24]	-19.6	27.65	25	-9.88	13.9	25	9.5%	-9.72 [-21.85, 2.41]
Soroka 1998 [25]	12	27.65	9	-11	13.9	9	8.7%	23.00 [2.78, 43.22]
**Subtotal (95% CI)**			**75**			**77**	**39.2%**	**-30.19 [-92.59, 32.21]**
Heterogeneity: Tau² = 4842.93; Chi² = 200.95, df = 4 (P < 0.00001); I² = 98%								
Test for overall effect: Z = 0.95 (P = 0.34)								
**Total (95% CI)**			**168**			**170**	**100.0%**	**-24.51 [-40.33, -8.69]**
Heterogeneity: Tau² = 645.04; Chi² = 278.73, df = 11 (P < 0.00001); I² = 96%								
Test for overall effect: Z = 3.04 (P = 0.002)								
Test for subgroup differences: Chi² = 0.13, df = 1 (P = 0.72), I² = 0%								

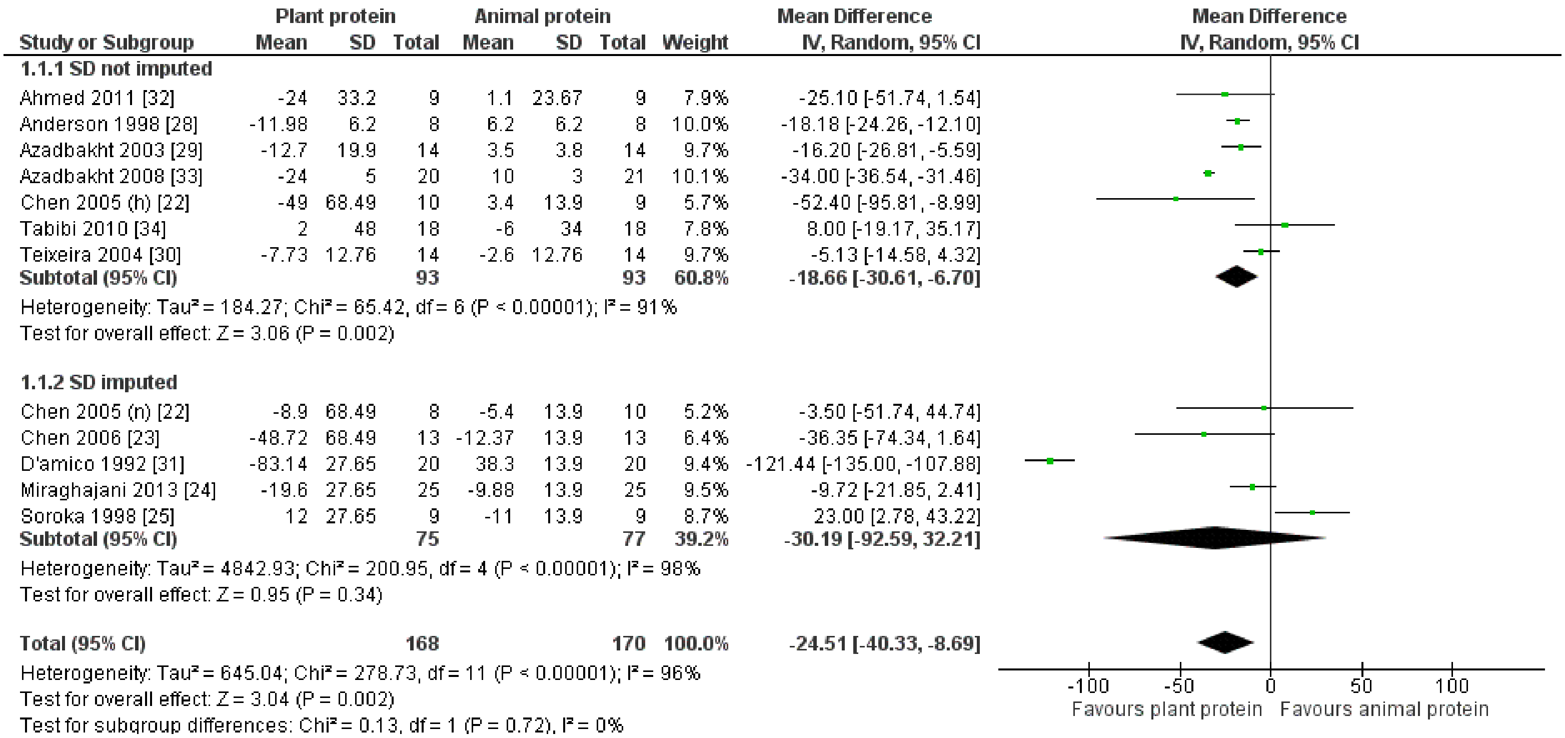

Figure S3. Meta-analysis of controlled trials assessing the effect of increased plant protein intake on total cholesterol levels of CKD patients according to the imputation of standard deviations

Study or Subgroup	Plant protein Mean	SD	Total	Animal protein Mean	SD	Total	Weight	Mean Difference IV, Random, 95% CI
**3.1.1 Crossover**								
Ahmed 2011 [32]	-63.6	203.79	9	-12.9	43.95	9		-50.70 [-186.90, 85.50]
Anderson 1998 [28]	-39.85	21.25	8	30.2	15	8	16.8%	-70.05 [-88.07, -52.03]
Azadbakht 2003 [29]	-9.9	12.9	14	3.2	3.7	14	22.6%	-13.10 [-20.13, -6.07]
Azadbakht 2008 [33]	-25	6	20	-6	2	21		-19.00 [-21.77, -16.23]
D'amico 1992 [31]	-32	93.84	20	-38	32.77	20	6.8%	6.00 [-37.56, 49.56]
Soroka 1998 [25]	14.9	93.84	9	-6.8	32.77	9	3.7%	21.70 [-43.24, 86.64]
Teixeira 2004 [30]	-4.4	46	14	-12.5	46	14	9.5%	8.10 [-25.98, 42.18]
**Subtotal (95% CI)**			**90**			**90**	**67.4%**	**-18.68 [-47.59, 10.23]**
Heterogeneity: Tau² = 984.71; Chi² = 39.69, df = 5 (P < 0.00001); I² = 87%								
Test for overall effect: Z = 1.27 (P = 0.21)								
**3.1.2 Parallel**								
Chen 2005 (h) [22]	-147.5	165	10	-35.5	32.77	9	1.6%	-112.00 [-216.48, -7.52]
Chen 2005 (n) [22]	-11.7	165	8	-4.9	32.77	10	1.3%	-6.80 [-122.93, 109.33]
Chen 2006 [23]	-20.37	165	13	-8	32.77	13	2.0%	-12.37 [-103.82, 79.08]
Miraghajani 2013 [24]	-39.64	93.84	25	-1.04	32.77	25		-38.60 [-77.56, 0.36]
Tabibi 2010 [34]	21	131	18	-14	86	18	3.0%	35.00 [-37.39, 107.39]
**Subtotal (95% CI)**			**78**			**80**	**32.6%**	**-18.54 [-37.42, 0.34]**
Heterogeneity: Tau² = 111.14; Chi² = 5.45, df = 5 (P = 0.36); I² = 8%								
Test for overall effect: Z = 1.92 (P = 0.05)								
**Total (95% CI)**			**168**			**170**	**100.0%**	**-21.88 [-35.35, -8.40]**
Heterogeneity: Tau² = 196.56; Chi² = 45.15, df = 11 (P < 0.00001); I² = 76%								
Test for overall effect: Z = 3.18 (P = 0.001)								
Test for subgroup differences: Chi² = 0.00, df = 1 (P = 0.99), I² = 0%								

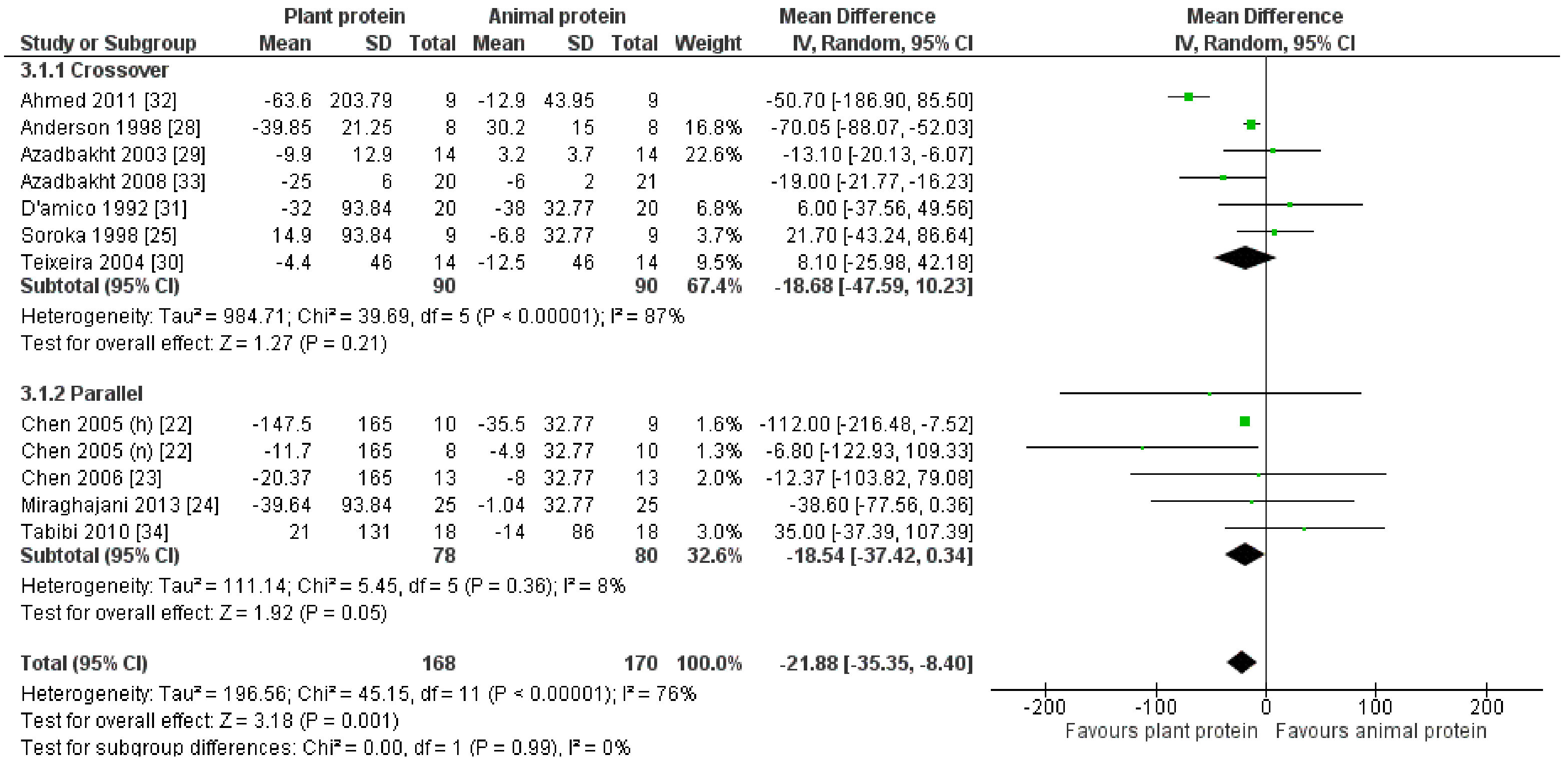

Figure S4. Meta-analysis of controlled trials assessing the effect of increased plant protein intake on triglycerides levels of CKD patients according to study design.

Study or Subgroup	Plant protein Mean	SD	Total	Animal protein Mean	SD	Total	Weight	Mean Difference IV, Random, 95% CI
**3.1.1 Some concerns about bias**								
Anderson 1998 [28]	-39.85	21.25	8	30.2	15	8	16.8%	-70.05 [-88.07, -52.03]
Azadbakht 2003 [29]	-9.9	12.9	14	3.2	3.7	14	22.6%	-13.10 [-20.13, -6.07]
Azadbakht 2008 [33]	-25	6	20	-6	2	21	23.8%	-19.00 [-21.77, -16.23]
Chen 2005 (h) [22]	-147.5	165	10	-35.5	32.77	9	1.6%	-112.00 [-216.48, -7.52]
Chen 2005 (n) [22]	-11.7	165	8	-4.9	32.77	10	1.3%	-6.80 [-122.93, 109.33]
Chen 2006 [23]	-20.37	165	13	-8	32.77	13	2.0%	-12.37 [-103.82, 79.08]
Soroka 1998 [25]	14.9	93.84	9	-6.8	32.77	9	3.7%	21.70 [-43.24, 86.64]
Teixeira 2004 [30]	-4.4	46	14	-12.5	46	14	9.5%	8.10 [-25.98, 42.18]
**Subtotal (95% CI)**			**96**			**98**	**81.2%**	**-24.35 [-39.53, -9.18]**
Heterogeneity: Tau² = 204.27; Chi² = 40.56, df = 7 (P < 0.00001); I² = 83%								
Test for overall effect: Z = 3.14 (P = 0.002)								
**3.1.2 High risk of bias**								
Ahmed 2011 [32]	-63.6	203.79	9	-12.9	43.95	9	0.9%	-50.70 [-186.90, 85.50]
D'amico 1992 [31]	-32	93.84	20	-38	32.77	20	6.8%	6.00 [-37.56, 49.56]
Miraghajani 2013 [24]	-39.64	93.84	25	-1.04	32.77	25	8.0%	-38.60 [-77.56, 0.36]
Tabibi 2010 [34]	21	131	18	-14	86	18	3.0%	35.00 [-37.39, 107.39]
**Subtotal (95% CI)**			**72**			**72**	**18.8%**	**-10.13 [-45.04, 24.78]**
Heterogeneity: Tau² = 394.47; Chi² = 4.37, df = 3 (P = 0.22); I² = 31%								
Test for overall effect: Z = 0.57 (P = 0.57)								
**Total (95% CI)**			**168**			**170**	**100.0%**	**-21.88 [-35.35, -8.40]**
Heterogeneity: Tau² = 196.56; Chi² = 45.15, df = 11 (P < 0.00001); I² = 76%								
Test for overall effect: Z = 3.18 (P = 0.001)								
Test for subgroup differences: Chi² = 0.54, df = 1 (P = 0.46), I² = 0%								

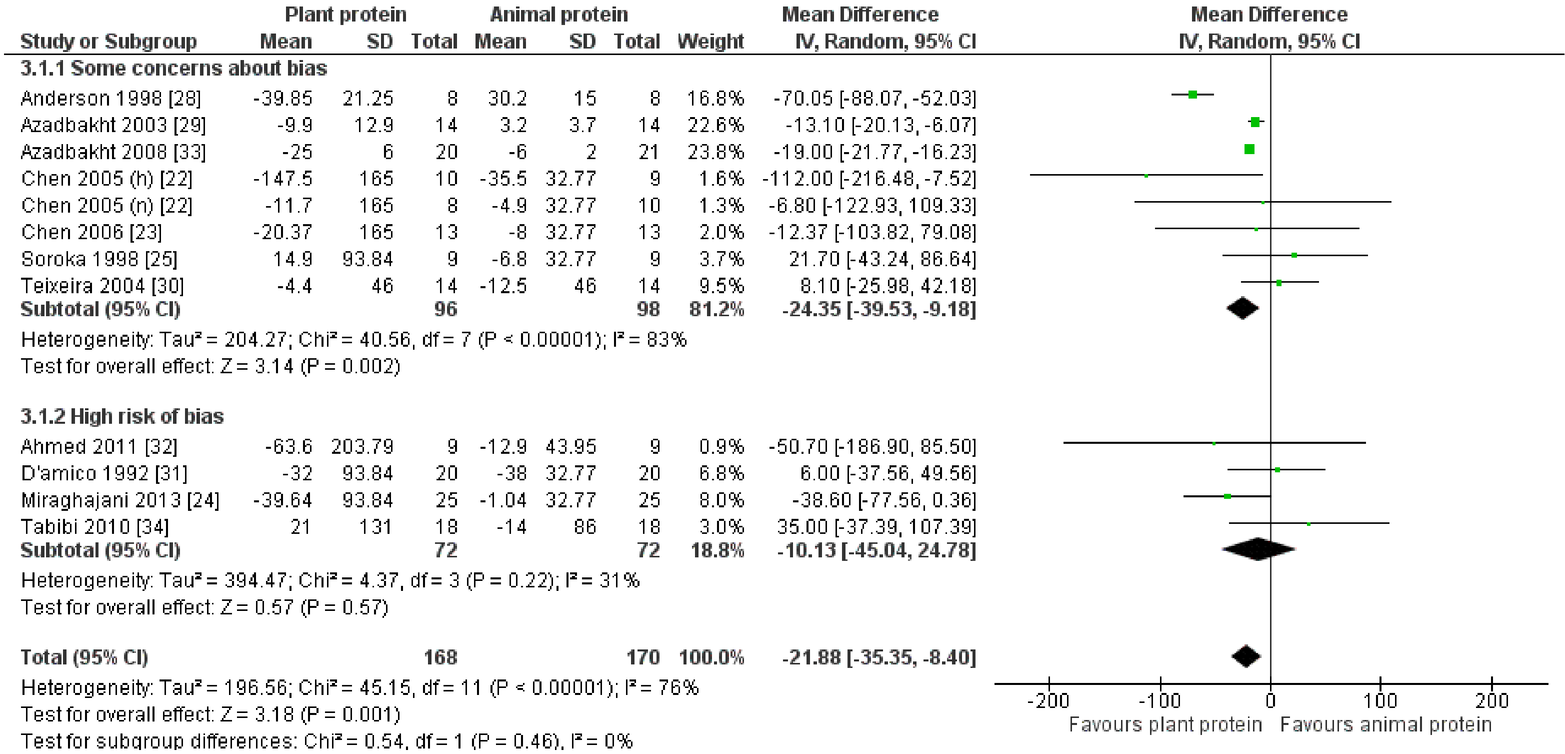

Figure S5. Meta-analysis of controlled trials assessing the effect of increased plant protein intake on triglycerides levels of CKD patients according to risk of bias.

Study or Subgroup	Plant protein Mean	Plant protein SD	Plant protein Total	Animal protein Mean	Animal protein SD	Animal protein Total	Weight	Mean Difference IV, Random, 95% CI
**3.1.1 SD not imputed**								
Ahmed 2011 [32]	-63.6	203.79	9	-12.9	43.95	9	0.9%	-50.70 [-186.90, 85.50]
Anderson 1998 [28]	-39.85	21.25	8	30.2	15	8	16.8%	-70.05 [-88.07, -52.03]
Azadbakht 2003 [29]	-9.9	12.9	14	3.2	3.7	14	22.6%	-13.10 [-20.13, -6.07]
Azadbakht 2008 [33]	-25	6	20	-6	2	21	23.8%	-19.00 [-21.77, -16.23]
Chen 2005 (h) [22]	-147.5	165	10	-35.5	32.77	9	1.6%	-112.00 [-216.48, -7.52]
Tabibi 2010 [34]	21	131	18	-14	86	18	3.0%	35.00 [-37.39, 107.39]
Teixeira 2004 [30]	-4.4	46	14	-12.5	46	14	9.5%	8.10 [-25.98, 42.18]
**Subtotal (95% CI)**			**93**			**93**	**78.2%**	**-25.13 [-40.96, -9.31]**
Heterogeneity: Tau² = 216.68; Chi² = 41.34, df = 6 (P < 0.00001); I² = 85%								
Test for overall effect: Z = 3.11 (P = 0.002)								
**3.1.2 SD imputed**								
Chen 2005 (n) [22]	-11.7	165	8	-4.9	32.77	10	1.3%	-6.80 [-122.93, 109.33]
Chen 2006 [23]	-20.37	165	13	-8	32.77	13	2.0%	-12.37 [-103.82, 79.08]
D'amico 1992 [31]	-32	93.84	20	-38	32.77	20	6.8%	6.00 [-37.56, 49.56]
Miraghajani 2013 [24]	-39.64	93.84	25	-1.04	32.77	25	8.0%	-38.60 [-77.56, 0.36]
Soroka 1998 [25]	14.9	93.84	9	-6.8	32.77	9	3.7%	21.70 [-43.24, 86.64]
**Subtotal (95% CI)**			**75**			**77**	**21.8%**	**-11.82 [-36.69, 13.06]**
Heterogeneity: Tau² = 0.00; Chi² = 3.49, df = 4 (P = 0.48); I² = 0%								
Test for overall effect: Z = 0.93 (P = 0.35)								
**Total (95% CI)**			**168**			**170**	**100.0%**	**-21.88 [-35.35, -8.40]**
Heterogeneity: Tau² = 196.56; Chi² = 45.15, df = 11 (P < 0.00001); I² = 76%								
Test for overall effect: Z = 3.18 (P = 0.001)								
Test for subgroup differences: Chi² = 0.78, df = 1 (P = 0.38), I² = 0%								

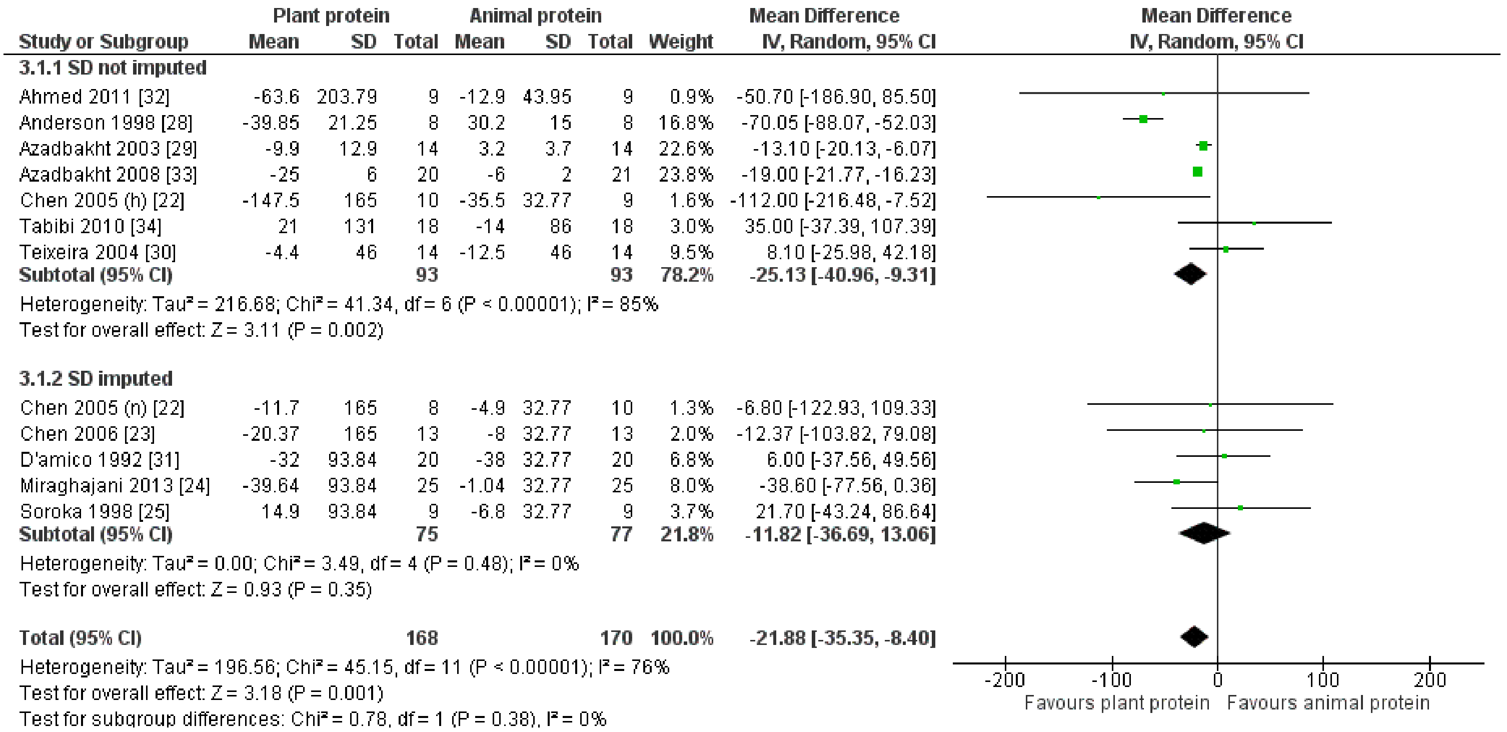

Figure S6. Meta-analysis of controlled trials assessing the effect of increased plant protein intake on triglycerides levels of CKD patients according to the imputation of standard deviations.

Study or Subgroup	Plant protein group Mean	Plant protein group SD	Plant protein group Total	Animal group Mean	Animal group SD	Animal group Total	Weight	Mean Difference IV, Random, 95% CI
**4.1.1 Some concerns about bias**								
Azadbakht 2003 [29]	-6.3	9.3	14	2	5.8	14	9.7%	-8.30 [-14.04, -2.56]
Azadbakht 2008 [33]	-21	5	20	7	2	21	9.8%	-28.00 [-30.35, -25.65]
Chen 2005 (h) [22]	-39.6	38.53	10	-8.9	9.66	9	8.1%	-30.70 [-55.40, -6.00]
Chen 2005 (n) [22]	-4.3	38.53	8	-5.1	9.66	10	7.8%	0.80 [-26.56, 28.16]
Chen 2006 [23]	-29.01	38.53	13	-4.26	9.66	13	8.4%	-24.75 [-46.34, -3.16]
Soroka 1998 [25]	-9	17.883	9	-5	9.66	9	9.3%	-4.00 [-17.28, 9.28]
Teixeira 2004 [30]	-2.4	8.5	14	0.3	8.5	14	9.7%	-2.70 [-9.00, 3.60]
**Subtotal (95% CI)**			**88**			**90**	**62.8%**	**-13.65 [-25.80, -1.50]**
Heterogeneity: Tau² = 209.25; Chi² = 93.14, df = 6 (P < 0.00001); I² = 94%								
Test for overall effect: Z = 2.20 (P = 0.03)								
**4.1.2 High risk of bias**								
Ahmed 2011 [32]	-31.9	22.68	9	4.8	17	9	8.8%	-36.70 [-55.22, -18.18]
D'amico 1992 [31]	-68.1	17.83	20	36.4	9.66	20	9.6%	-104.50 [-113.39, -95.61]
Miraghajani 2013 [24]	-15.48	17.83	25	-17	9.66	25	9.6%	1.52 [-6.43, 9.47]
Tabibi 2010 [34]	-0.5	23	18	-1	15	18	9.3%	0.50 [-12.19, 13.19]
**Subtotal (95% CI)**			**72**			**72**	**37.2%**	**-34.83 [-93.17, 23.51]**
Heterogeneity: Tau² = 3502.51; Chi² = 345.36, df = 3 (P < 0.00001); I² = 99%								
Test for overall effect: Z = 1.17 (P = 0.24)								
**Total (95% CI)**			**160**			**162**	**100.0%**	**-21.71 [-38.32, -5.10]**
Heterogeneity: Tau² = 730.63; Chi² = 466.77, df = 10 (P < 0.00001); I² = 98%								
Test for overall effect: Z = 2.56 (P = 0.01)								
Test for subgroup differences: Chi² = 0.49, df = 1 (P = 0.49), I² = 0%								

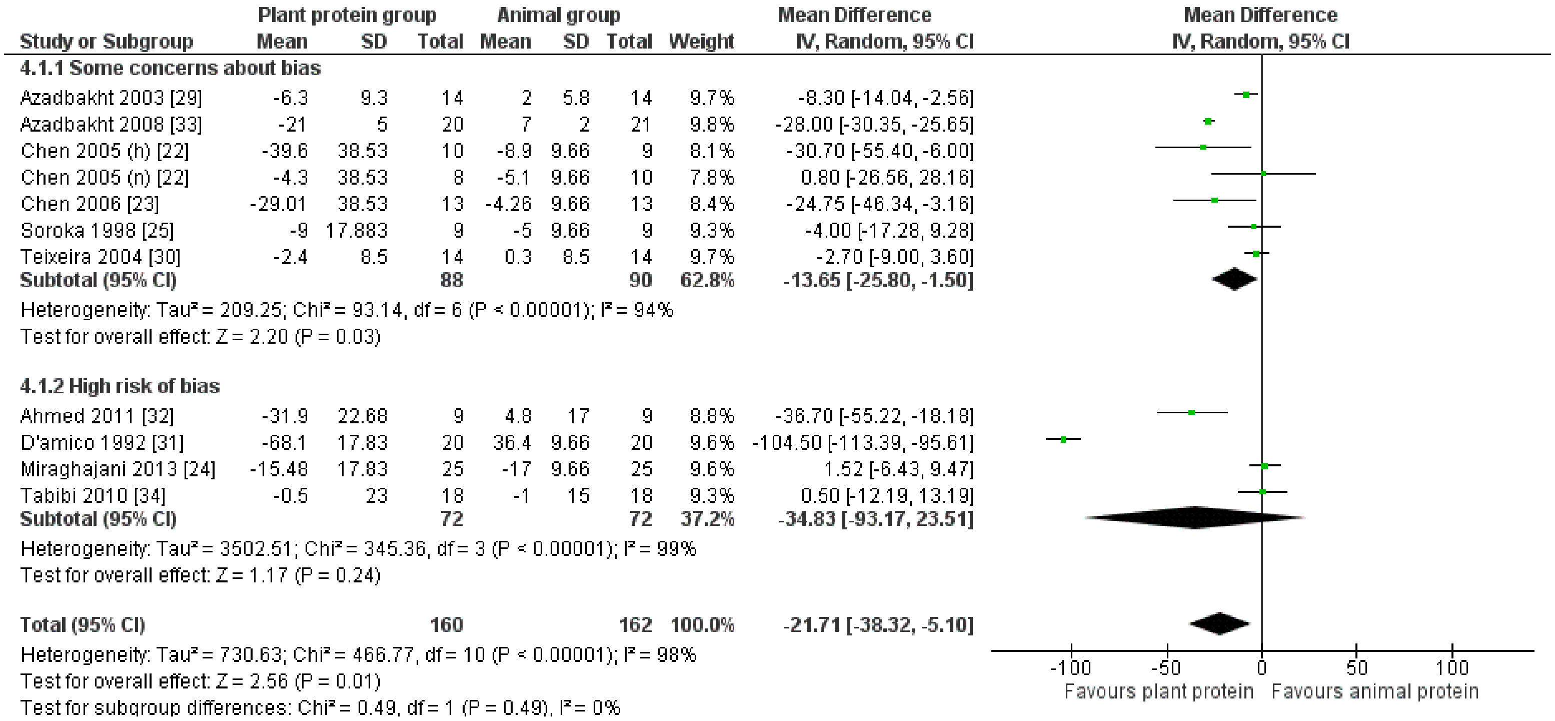

Figure S7. Meta-analysis of controlled trials assessing the effect of increased plant protein intake on LDL levels of CKD patients according to the risk of bias.

Study or Subgroup	Plant protein group Mean	SD	Total	Animal group Mean	SD	Total	Weight	Mean Difference IV, Random, 95% CI
**4.1.1 SD not imputed**								
Ahmed 2011 [32]	-31.9	22.68	9	4.8	17	9	8.8%	-36.70 [-55.22, -18.18]
Azadbakht 2003 [29]	-6.3	9.3	14	2	5.8	14	9.7%	-8.30 [-14.04, -2.56]
Azadbakht 2008 [33]	-21	5	20	7	2	21	9.8%	-28.00 [-30.35, -25.65]
Chen 2005 (h) [22]	-39.6	38.53	10	-8.9	9.66	9	8.1%	-30.70 [-55.40, -6.00]
Tabibi 2010 [34]	-0.5	23	18	-1	15	18	9.3%	0.50 [-12.19, 13.19]
Teixeira 2004 [30]	-2.4	8.5	14	0.3	8.5	14	9.7%	-2.70 [-9.00, 3.60]
**Subtotal (95% CI)**			**85**			**85**	**55.4%**	**-16.25 [-29.27, -3.23]**
Heterogeneity: Tau² = 222.93; Chi² = 97.78, df = 5 (P < 0.00001); I² = 95%								
Test for overall effect: Z = 2.45 (P = 0.01)								
**4.1.2 SD imputed**								
Chen 2005 (n) [22]	-4.3	38.53	8	-5.1	9.66	10	7.8%	0.80 [-26.56, 28.16]
Chen 2006 [23]	-29.01	38.53	13	-4.26	9.66	13	8.4%	-24.75 [-46.34, -3.16]
D'amico 1992 [31]	-68.1	17.83	20	36.4	9.66	20	9.6%	-104.50 [-113.39, -95.61]
Miraghajani 2013 [24]	-15.48	17.83	25	-17	9.66	25	9.6%	1.52 [-6.43, 9.47]
Soroka 1998 [25]	-9	17.883	9	-5	9.66	9	9.3%	-4.00 [-17.28, 9.28]
**Subtotal (95% CI)**			**75**			**77**	**44.6%**	**-26.48 [-78.51, 25.54]**
Heterogeneity: Tau² = 3444.36; Chi² = 344.06, df = 4 (P < 0.00001); I² = 99%								
Test for overall effect: Z = 1.00 (P = 0.32)								
**Total (95% CI)**			**160**			**162**	**100.0%**	**-21.71 [-38.32, -5.10]**
Heterogeneity: Tau² = 730.63; Chi² = 466.77, df = 10 (P < 0.00001); I² = 98%								
Test for overall effect: Z = 2.56 (P = 0.01)								
Test for subgroup differences: Chi² = 0.14, df = 1 (P = 0.71), I² = 0%								

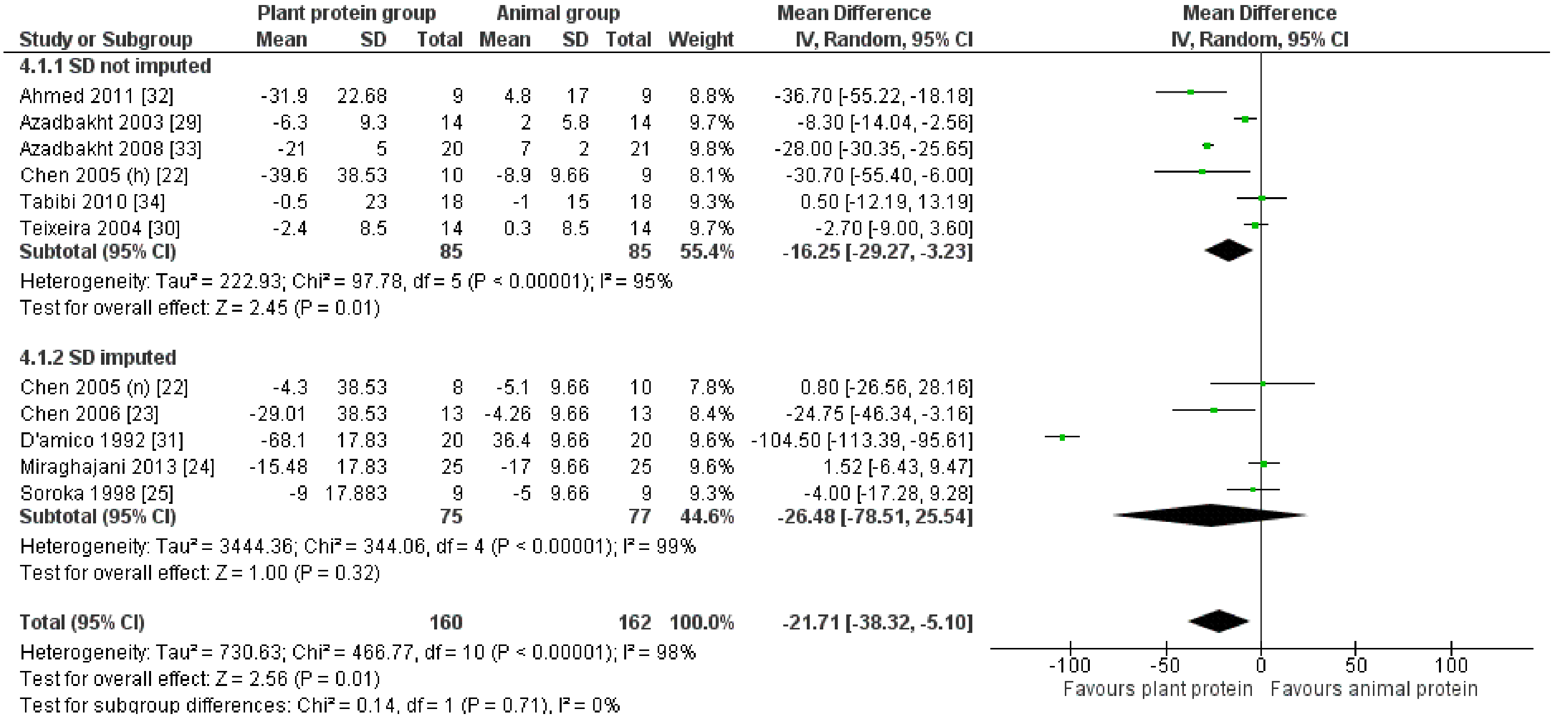

Figure S8. Meta-analysis of controlled trials assessing the effect of increased plant protein intake on LDL levels of CKD patients according to the imputation of standard deviations.

Study or Subgroup	Plant protein group Mean	SD	Total	Animal protein group Mean	SD	Total	Weight	Mean Difference IV, Fixed, 95% CI
**5.1.1 Some concerns about bias**								
Azadbakht 2003 [29]	49.1	12.6	14	46.4	13.5	14	3.9%	2.70 [-6.97, 12.37]
Azadbakht 2008 [33]	53	31	20	45	19	21	1.4%	8.00 [-7.83, 23.83]
Chen 2005 (h) [22]	39.1	7.6	10	36.8	5.7	9	10.1%	2.30 [-3.70, 8.30]
Chen 2005 (n) [22]	36.5	11.2	8	37.8	7	10	4.6%	-1.30 [-10.19, 7.59]
Chen 2006 [23]	39.83	7.34	13	37.9	5	13	15.6%	1.93 [-2.90, 6.76]
Soroka 1998 [25]	38.6	4	9	41.4	3	9	34.0%	-2.80 [-6.07, 0.47]
Teixeira 2004 [30]	38.6	7.2	14	34.4	8.7	14	10.4%	4.20 [-1.72, 10.12]
**Subtotal (95% CI)**			**88**			**90**	**79.8%**	**0.22 [-1.91, 2.35]**
Heterogeneity: Chi² = 7.26, df = 6 (P = 0.30); I² = 17%								
Test for overall effect: Z = 0.20 (P = 0.84)								
**5.1.2 High risk of bias**								
Ahmed 2011 [32]	60	20.3	9	54.6	14.1	9	1.4%	5.40 [-10.75, 21.55]
D'amico 1992 [31]	47.56	15.85	20	57.23	20.1	20	2.9%	-9.67 [-20.89, 1.55]
Miraghajani 2013 [24]	44.12	9.25	25	43.56	11.8	25	10.5%	0.56 [-5.32, 6.44]
Tabibi 2010 [34]	43	9.5	18	42	15	18	5.4%	1.00 [-7.20, 9.20]
**Subtotal (95% CI)**			**72**			**72**	**20.2%**	**-0.45 [-4.69, 3.79]**
Heterogeneity: Chi² = 3.33, df = 3 (P = 0.34); I² = 10%								
Test for overall effect: Z = 0.21 (P = 0.84)								
**Total (95% CI)**			**160**			**162**	**100.0%**	**0.09 [-1.82, 1.99]**
Heterogeneity: Chi² = 10.67, df = 10 (P = 0.38); I² = 6%								
Test for overall effect: Z = 0.09 (P = 0.93)								
Test for subgroup differences: Chi² = 0.08, df = 1 (P = 0.78), I² = 0%								

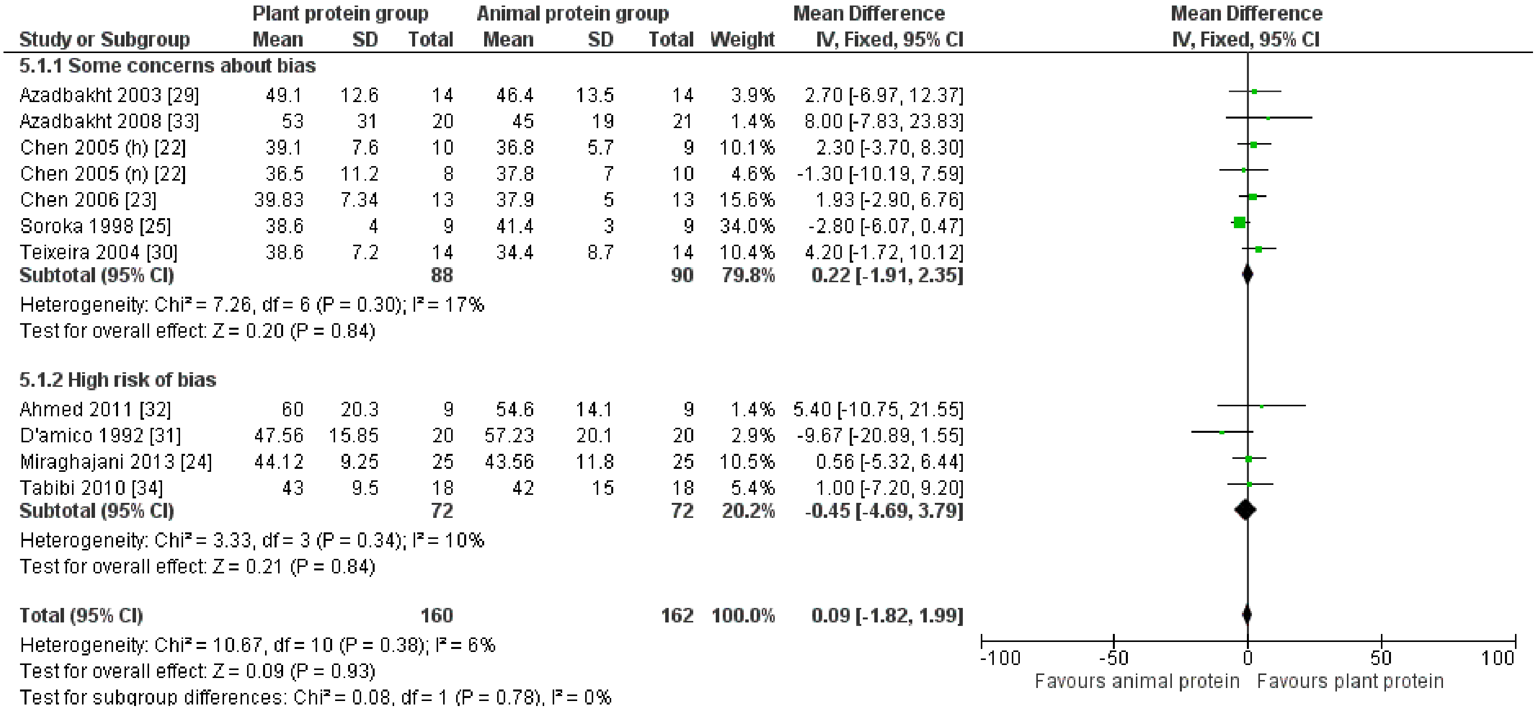

Figure S9. Meta-analysis of controlled trials assessing the effect of increased plant protein intake on HDL levels of CKD patients according to the risk of bias.

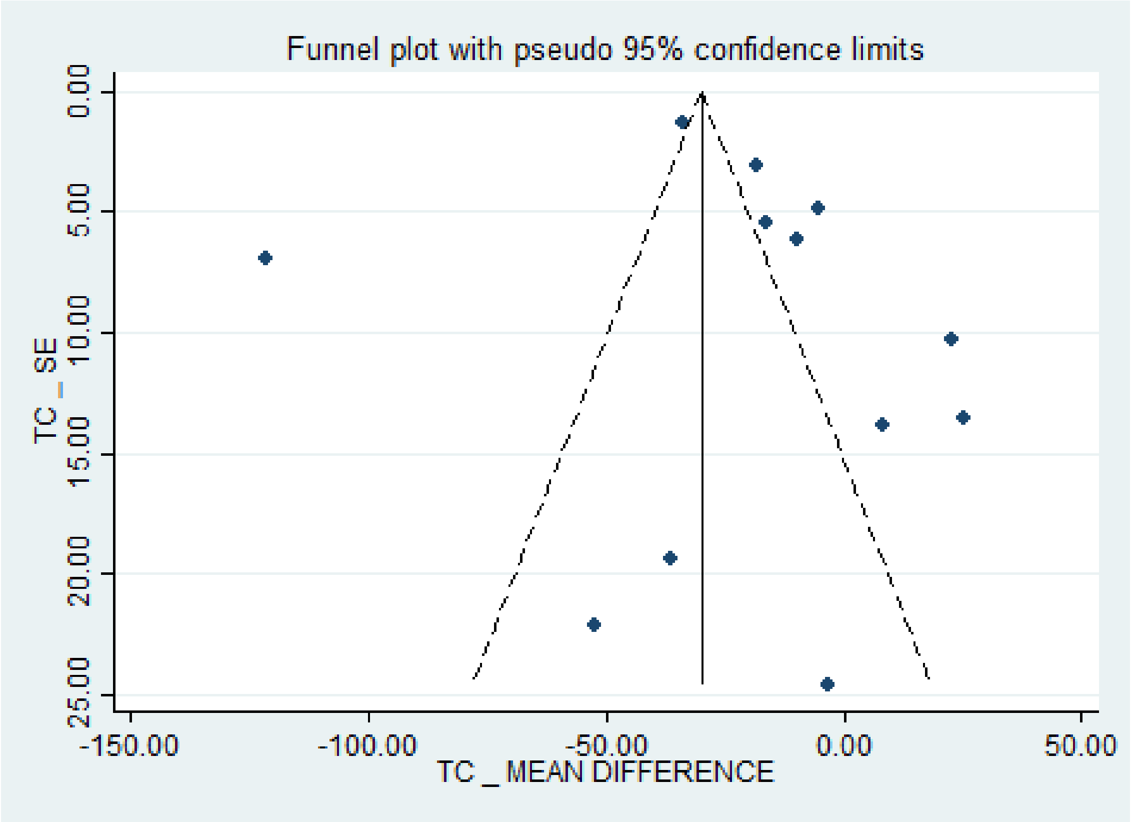

Figure S10. Funnel plot of the standard errors (SE) versus mean differences (MD) of total cholesterol levels.

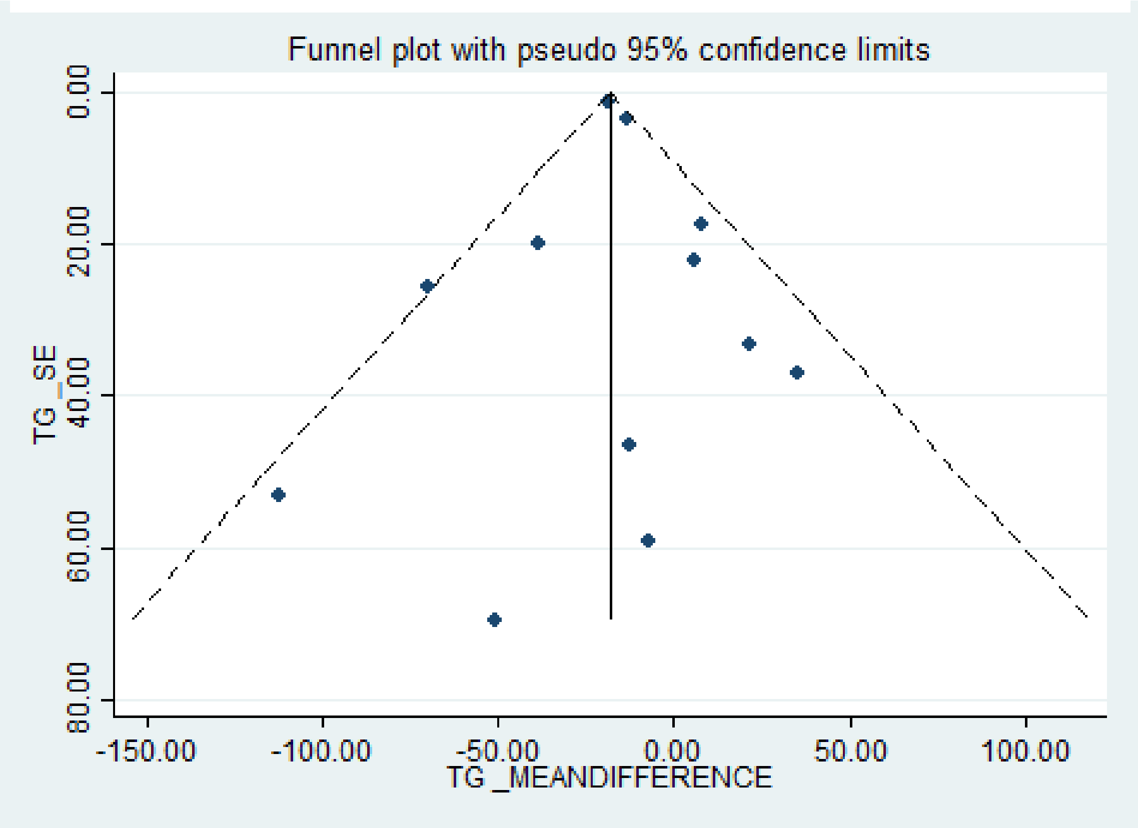

Figure S11. Funnel plot of SE versus MD of triglycerides levels

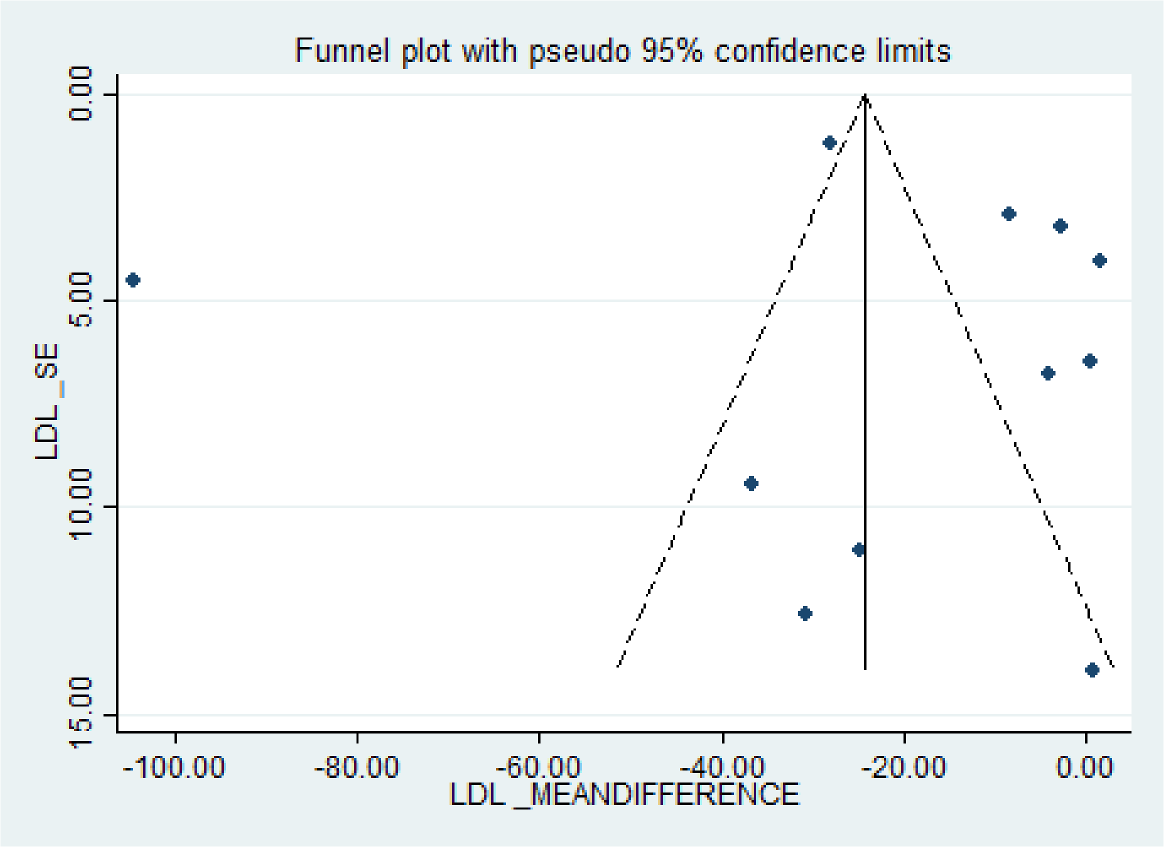

Figure S12. Funnel plot of SE versus MD of LDL cholesterol levels.

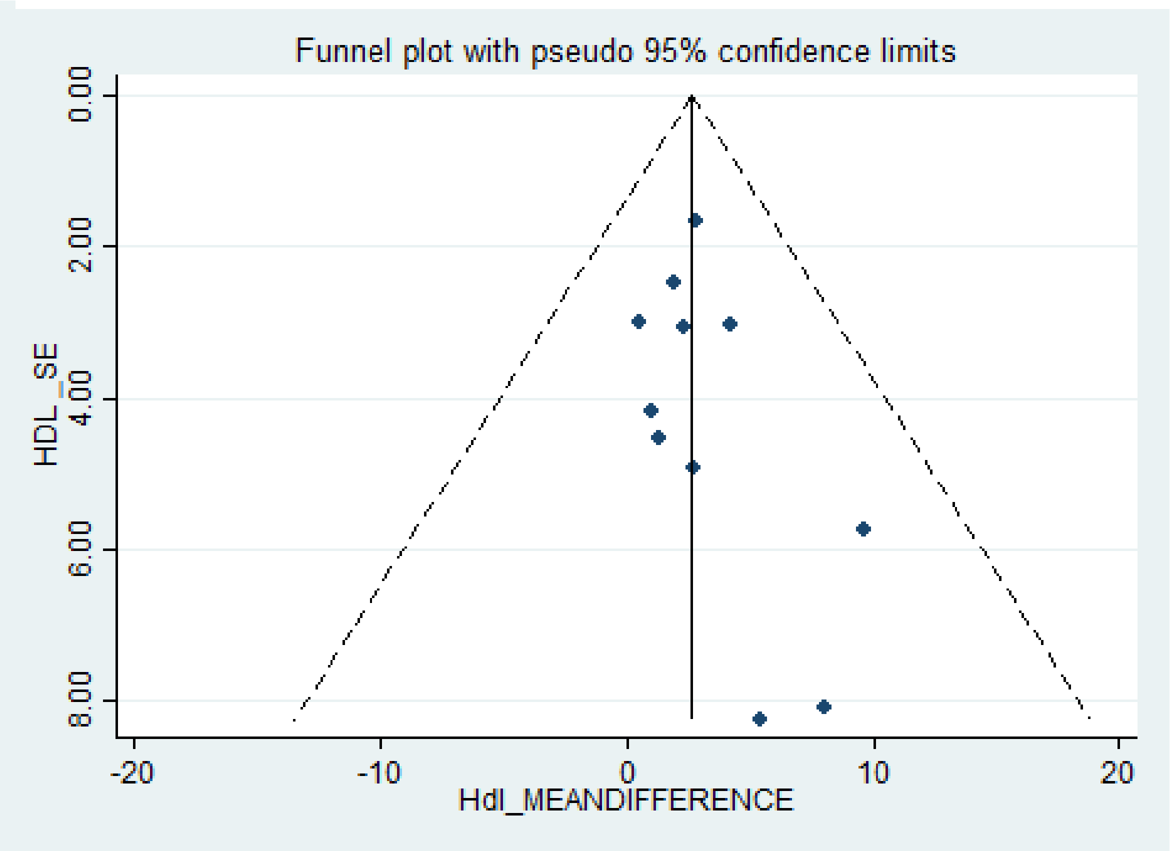

Figure S13. Funnel plot of SE versus MD of HDL cholesterol levels.
